# Alginate oligosaccharides improve germ cell development and testicular microenvironment to rescue busulfan disrupted spermatogenesis

**DOI:** 10.7150/thno.43189

**Published:** 2020-02-10

**Authors:** Yong Zhao, Pengfei Zhang, Wei Ge, Yanni Feng, Lan Li, Zhongyi Sun, Hongfu Zhang, Wei Shen

**Affiliations:** 1 College of Life Sciences, Qingdao Agricultural University, Qingdao 266109, P. R. China.; 2 State Key Laboratory of Animal Nutrition, Institute of Animal Sciences, Chinese Academy of Agricultural Sciences, Beijing 100193, P. R. China; 3 College of Veterinary Sciences, Qingdao Agricultural University, Qingdao 266109, P. R. China.; 4 Center for Reproductive Medicine, Urology Department, Peking University Shenzhen Hospital, Shenzhen 518036, P. R. China.

**Keywords:** AOS, Single cell RNA sequencing, Spermatogenesis, Metabolome, Microbiota

## Abstract

**Rationale**: Busulfan is currently an indispensable anti-cancer drug, particularly for children, but the side effects on male reproduction are so serious that critical drug management is needed to minimize any negative impact. Meanwhile, alginate oligosaccharides (AOS) are natural products with many consequent advantages, that have attracted a great deal of pharmaceutical attention. In the current investigation, we performed single-cell RNA sequencing on murine testes treated with busulfan and/or AOS to define the mitigating effects of AOS on spermatogenesis at the single cell level.

**Methods**: Testicular cells (*in vivo*) were examined by single cell RNA sequencing analysis, histopathological analysis, immunofluorescence staining, and Western blotting. Testes samples (*ex vivo*) underwent RNA sequencing analysis. Blood and testicular metabolomes were determined by liquid chromatography-mass spectrometry (LC/MS).

**Results**: We found that AOS increased murine sperm concentration and motility, and rescued busulfan disrupted spermatogenesis through improving (i) the proportion of germ cells, (ii) gene expression important for spermatogenesis, and (iii) transcriptional factors *in vivo*. Furthermore, AOS promoted the *ex vivo* expression of genes important for spermatogenesis*.* Finally, our results showed that AOS improved blood and testis metabolomes as well as the gut microbiota to support the recovery of spermatogenesis.

**Conclusions**: AOS could be used to improve fertility in patients undergoing chemotherapy and to combat other factors that induce infertility in humans.

## Introduction

Spermatozoa develops from spermatogonial stem cells (SSC) in the testis during the complex and orchestrated process of spermatogenesis. During spermatogenesis, spermatogonia (SPGs) develop into spermatocytes (SPCs); then, following meiosis, haploid spermatids (STs) are formed which subsequently develop into spermatozoa 1,2. Spermatogonial stem cells (SSCs) are a group of undifferentiated SPGs that can be self-renewing and are responsible for maintaining the pool of male germ cells. Somatic cells, namely Sertoli cells (SCs) and Leydig cells (LCs) play indispensable roles in spermatogenesis. SCs act as nurse cells to maintain germ cell differentiation, whereas LCs produce androgens and other factors to support spermatogenesis. Another type of somatic cell, peritubular myoid cells (PTMs) are muscle cells that support the seminiferous tubules. All these cells orchestrate together to produce functional sperm 1-3. This process, however, is vulnerable to environmental contamination, anti-cancer drugs, and other factors 3-7.

For many reasons, the incidence of malignancies continues to rise worldwide and has resulted in the increased use of anti-cancer drugs 8-10. Busulfan is an anti-cancer alkylating agent that has been widely used to treat lymphoma, chronic leukemia, and is also used to improve outcomes after allogeneic haemopoietic cell transplantation or the conditioning regimen before hematopoietic stem cell transplantation 11-13. Busulfan is one of the very few anti-cancer drugs used in children under the age of three 12-14; it gives excellent results but it also produces several side effects 11,13, one being its toxicity on the reproductive system 15. Busulfan can destroy testicular germ cells, decrease testis weight and sperm motility, increase sperm abnormalities and oligo-azoospermia rate, and finally cause temporary or permanent sterility 8,15,16. Since busulfan is such an important anti-cancer drug, especially for children, and the side effects on male reproduction are so serious, it is critical to employ good drug management to minimize its negative impact. Many studies report on various management tools that have been used in an attempt to reduce the side effects of busulfan on male reproduction, particularly spermatogenesis 5-7. Molybdenum (Mo) can rescue germ cell development, and maintain blood testosterone, estradiol, and luteinizing hormone levels to improve spermatogenesis in mice 6. Olive leaf extract ameliorates the negative effect of busulfan on spermatogenesis 8. Korean red ginseng has been found to attenuate busulfan induced disruption of spermatogenesis 5. Furthermore, Chi et al. report that genistein can decrease intra-testicular testosterone (ITT) levels and improve spermatogenesis in rats after busulfan treatment, which indicates that it could potentially rescue male fertility in busulfan treated cancer patients 7.

Alginate oligosaccharides (AOS) have attracted considerable pharmaceutical attention due to their natural properties, which include non-immunogenicity, non-toxicity, and biodegradability (biodegradable polymers) 17-22. AOS are the degradation products of alginate (one type of marine polysaccharide from brown seaweed) which are composed of α-L-guluronate (G) and β-D-mannuronate (M) joined by 1, 4-glycoside bonds 18. AOS have many pharmacological benefits in the fields of anti-inflammation 19, anti-apoptosis 23, anti-proliferation 24, antioxidant activities 22,23,25, and even anti-cancer properties 26. AOS decrease the formation of nitric oxide and prostaglandin E2, reduce the secretion of proinflammatory cytokines, and inhibit the expression of toll-like receptor 4, nuclear factor (NF)-κB, inducible nitric oxide synthase, and cyclooxygenase-2 to prevent lipopolysaccharide /β-amyloid (Aβ)-induced neuroinflammation 27. It has been demonstrated that AOS protects against acute cardiotoxicity [myocardial ischemia/reperfusion (I/R) injury] produced by the highly potent chemotherapeutic agent doxorubicin through blocking oxidative stress and endoplasmic reticulum stress-mediated apoptosis 25,28. Tusi et al., discovered that AOS can prevent neurodegenerative diseases (Alzheimer's disease) similarly through anti-oxidant and anti-ER stress-induced apoptosis 23. Because of these qualities, AOS have been recognized as a safe biopolymer by the U.S. Food and Drug Administration (reference no. 21CFR184.1724) and have been used in pharmaceutical, cosmeceutical, and nutraceutical fields 18,29. Although many efforts have been made to improve spermatogenesis after disruption by busulfan, there has been scant progress and little understanding of the improvement of spermatogenesis at the single cell level. Nowadays, however, single cell RNA sequencing analysis (scRNA-seq) can be used to understand human spermatogenesis at the single cell level 1,2. In this investigation, we performed scRNA-seq on murine testes treated with busulfan and/or AOS to define the rescuing effect of AOS on spermatogenesis at the single cell level. By comparing gene expression profiles of single cells from busulfan treated or busulfan plus AOS treated murine testes, we found that AOS can rescue busulfan disrupted spermatogenesis.

## Results

### AOS Increased the Motility and Concentration of Mouse Spermatozoa

**Figure 1:**
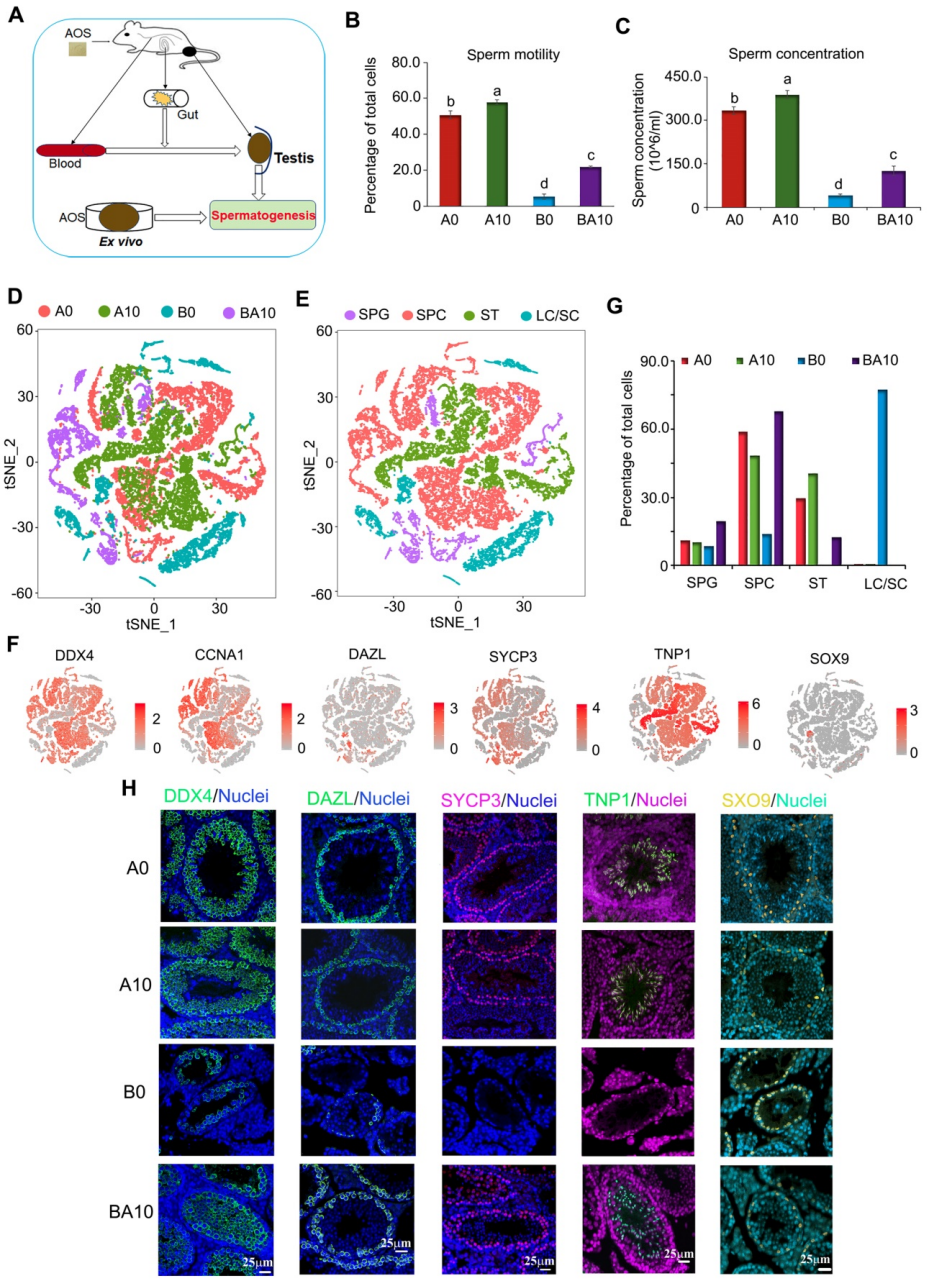
** Mouse sperm motility, concentration, and scRNA-seq analysis.** (**A**) Study design. (**B**) Mouse sperm motility. The y-axis represents the percentage of cells. The X-axis represents the treatment (n = 30/group). ^a,b,c^ Means not sharing a common superscript are different (*p <* 0.05). (**C**) Mouse sperm concentration. The y-axis represents the concentration. The x-axis represents the treatment (n = 30/group). ^a,b,c^ Means not sharing a common superscript are different (*p <* 0.05). (**D**) scRNA-seq cell map based on tSNE for the four treatment groups. (**E**) Cell clusters in scRNA-seq analysis. (**F**) Marker genes for each cluster. (**G**) The proportion of cells in each cluster in every sample. (**H**) IHF for some of the marker genes.

As shown in Figure 1A, three-week-old ICR male mice were treated with busulfan once (40 mg/Kg body weight, BW) 5. The following day, the mice were dosed with a vehicle control (ddH_2_O) or AOS in ddH_2_O for five weeks. There were four treatment groups: A0 (vehicle control, ddH_2_O alone), A10 (10 mg/kg BW of AOS), B0 (busulfan alone)), and BA10 (busulfan plus AOS 10 mg/kg BW). Tissues were prepared as follows: sperm motility and concentration were determined by computer-assisted sperm assay (CASA), testis tubular cells were collected for 10x scRNA-seq, intestinal digesta was collected for 16s analysis, plasma was used to determine changes in blood metabolism, and testicular tissue was homogenized, after which the supernatant was used to determine alterations in testicular metabolism. We found that busulfan diminished murine sperm motility and concentration (Figure 1B-C). Compared to A0, A10 significantly increased sperm motility (113.8%) and concentration (116.8%), although the increase was relatively slight. However, compared to B0, BA10 dramatically increased sperm motility (4.2-fold) and concentration (3.1-fold; Figure 1B-C). The data were consistent with the testicular histopathology (Figure S1A).

### Global Transcriptional Profiling by scRNA-seq Analysis Revealed that AOS Rescued Busulfan Disrupted Spermatogenesis *in vivo*

After five weeks of treatment, murine testes were resected and digested, and single cells were collected for the 10x scRNA-seq analysis. In total, 8941, 9682, 4659, and 3778 testicular cells were obtained for A0, A10, B0, and BA10 treatment groups, respectively, using scRNA-seq analysis (Figure S1B). The average number of genes and unique molecular identifiers (UMI) per cell are presented in Figure S1C.

After filtration, data from the four groups were analyzed together using the Seurat package in R studio. t-distributed stochastic neighbor embedding (t-SNE) analysis was used to identify the cell types (Figure 1D; Figure S1D; Table S1) 1,2,30. In total, 27 060 cells from the four groups were organized into 16 clusters (Figure S1E). The marker genes for testicular germ cells and somatic cells have been reported previously 1-3. Using these marker genes in the current study, the 16 cell clusters were placed into four major groups: SPGs, SPCs, STs, and LCs/SCs (Figure 1E; Table S2). Other types of somatic cells were not found in our data as only testis tubular cells were collected for sequencing. The marker genes for SPGs are STK31, DAZL, ID4, NOC4L, and PTRHD1 which were present in clusters 8, 9, and 10; these three clusters were placed into one big cluster, namely SPGs (Figure 1E-F; Figure S1F). The marker genes for SPCs are SYCP1, SYCP3, REC8, and SPO11, which were found to be expressed mainly in clusters 0, 2, 3, 6, 13, 14, and 15; these seven clusters were grouped into one big cluster, namely SPCs (Figure 1E-F; Figure S1F). The marker genes for STs are PRM1, PRM23, TNP1, and TNP2, which were mainly expressed in clusters 1, 4, and 7; these three clusters were placed into one big cluster, namely STs (Figure 1E-F; Figure S1F). The marker genes for LCs are HSD3B1, STAR, INSL3 and the marker gene for SCs is SOX9, which were mainly expressed in clusters 5, 11, and 12 with SC count being low throughout. These three clusters (5, 11, and 12) were organized into one big cluster: LCs/SCs (Figure 1E-F; Figure S1F).

In the four large clusters (SPGs, SPCs, STs, and LCs/SCs) the percentage of each type of cell for each treatment group (A0, A10, B0, and BA10) were calculated (Figure 1G). Similar percentages of cells were found in the SPGs cluster between A0 and A10 (10.98% vs. 10.28%). The percentage of cells in the SPGs cluster was lowest (8.50%) for B0, and highest for BA10 (19.64%). The percentages of cells in the SPCs cluster were 58.92%, 48.46%, 13.87%, and 67.87% for A0, A10, B0, and BA10, respectively; while the percentages of cells in the STs cluster were 29.48%, 40.67%, 0.36%, and 12.44%, respectively. However, the percentages of cells in the LCs/SCs cluster were 0.62%, 0.59%, 77.27%, and 0.05%, respectively. This data showed no notable difference for all four treatment groups in the SPGs cluster; however, the percentage of SPCs and STs were much lower in the busulfan treatment group. Results indicated that AOS rescued these levels by dramatically increasing the number of SPCs and STs in B+A 10.

Furthermore, some of these proteins for the marker genes (DDX4, DAZL, SYCP3, TNP1, and SOX9) 1-3 were also determined in the testis samples by immunofluorescence staining (IHF; Figure 1H; Figure S2A-D). Busulfan (B0) decreased the number of DDX4 positive cells, whereas BA10 significantly increased them (Figure 1H, Figure S2A). DAZL is the SPG marker gene 1-3 and DAZL protein is expressed in SPGs (Figure 1H). The percentage of DAZL positive cells was lowest in B0; however, it was increased in BA10, to almost similar levels as in A0 or A10 (Figure 1H and Figure S2B). SYCP3 is the SPC marker gene, it is a synaptonemal complex protein that plays a vital role in meiosis 1-3 and is expressed in early SPCs (Figure 1H). SYCP3 positive cells were scarce in the B0 group; however, they were present in similar numbers in BA10, A0, and A10 (Figure 1H; Figure S2C). TNP1 is the ST marker gene 1-3 and is expressed in STs (Figure 1H). Similar to DAZL and SYCP3, TNP1 positive cells were present at their lowest numbers in the B0 group; meanwhile, they were increased in BA10 and to similar levels as in A0 and A10 (Figure 1H; Figure S2D). SOX9 is the SC marker gene 1-3 and is expressed in SCs (Figure 1H). Because there were so few germ cells in the sample of the B0 group, the protein levels of SOX9 seemed to be brighter; however, the number of SCs were similar for all groups. The IHF data and scRNA-seq data concurred for these four treatment groups. The data suggested that busulfan disrupted the process of spermatogenesis. However, AOS protected spermatogenesis by promoting the development of spermatogonia to spermatocytes and on to spermatids.

### Deep Analysis of 10x scRNA-seq Data Discovered that AOS Improved Germ Cell Development Potential and Functions *in vivo*

**Figure 2:**
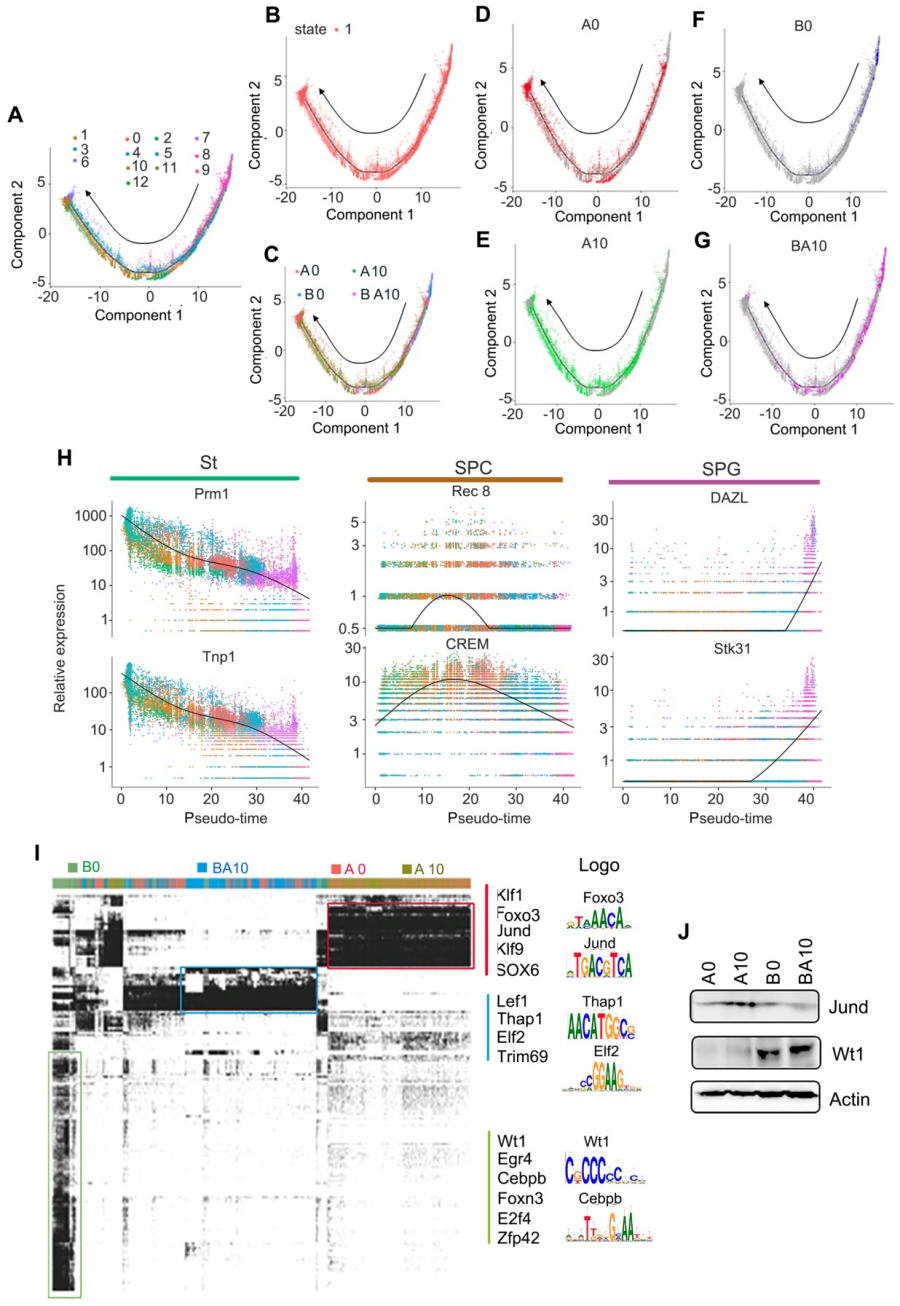
** Pseudotime analysis and transcriptional factor screening for scRNA-seq data.** (**A**) Trajectory reconstruction of SPGs, SPCs, and STs based on cell clusters. (**B**) State status in pseudotime analysis. (**C**) Trajectory plot based on different treatment samples. (**D**) Trajectory plot for AOS 0. (**E**) Trajectory plot for AOS 10. (**F**) Trajectory plot for B+A 0. (**G**) Trajectory plot for B+A 10. (**H**) Marker gene expression patterns in Monocle analysis. (**I**) SCENIC results from the murine testis samples. Different transcriptional factors in different samples AOS 0, AOS 10, B+A 0, and B+A 10. (**J**) WB data for some of the transcriptional factors.

In order to confirm the above data, we isolated germ cells (SPGs, SPCs, and STs clusters) from the total cell population. These germ cells were divided into 13 clusters by using similar criteria as for whole population cell analysis (Figure S2E-F). The marker genes for these clusters were identified and showed similar trends to the whole population analysis (Figure S2G-J). Subsequently, we performed Monocle analysis to determine the temporal order of these germ cells (SPGs, SPCs, and STs clusters). The unsupervised pseudotime, from SPGs to SPCs to STs, showed the developmental trajectory of mouse spermatogenesis (Figure 2A). We also checked the state of cells using Monocle analysis, and found that these cells were all at one developmental stage, which indicated that the cells matured in a strict time order (Figure 2B). We also checked the cells in each sample (A0, A10, B0, BA10) in the pseudotime plots (Figure 2C-G); we found that the cells for the A0 and A10 groups were similar (Figure 2D-E). However, there were only a few cells in the early stages of development (SPGs), and almost no cells in the SPC and ST stages in the B0 group (Figure 2F). On the other hand, there were many cells in the SPG and SPC stages and also some at ST in the pseudotime plot of the BA10 group (Figure 2G). Furthermore, we found that these three clusters (SPGs, SPCs, and STs) could be distinguished from each other by a few genes (Figure 2H). Based on subsequent t-distributed stochastic neighbor embedding (t-SNE) analysis, we found that there were specific differentially expressed marker genes for each cluster (SPGs, SPCs, STs, and LCs/SCs). The expression levels of the first 50 marker genes in each cluster are shown in Figure S3A-D. The data further suggested that AOS can protect spermatogenesis by regulating the expression of important genes.

Furthermore, in order to deeply investigate the mechanism by which AOS rescues spermatogenesis, gene regulatory networks were constructed using the single-cell regulatory network inference and clustering (SCENIC) computational pipeline 31. Germ cell identity and cell fate are governed by transcription factors and associated cofactors and they work in a coordinated manner to regulate the expression of target genes. SCENIC is an excellent tool for mapping the gene regulatory network (GRN) 31. The SCENIC AUCell algorithm was used in this study for cells in the SPG, SPC, and ST clusters 31. In total, 185 regulons were identified, differentially expressed, and were active in the three treatment groups of testicular samples (Figure 2I; Table S3, Table S4). The active regulons were sample specific in each group (Figure 2I). Some of the important regulons were specifically expressed in A0 and A10, such as Klf1, Jund, and Sox6. Meanwhile some regulons were specifically expressed in BA10, such as Lef1 and Elf2, and some regulons were specifically expressed in B0, such as Wt1 and Egr4. The protein levels of some regulons were explored by western blotting (WB) (Figure 2J). We found that Jund was more abundant in the A10 group while Wt1 was more abundant in BA10 (Figure 2J). This data indicated that AOS modified transcription factors to regulate gene expression to, in turn, rescue spermatogenesis.

**Figure 3:**
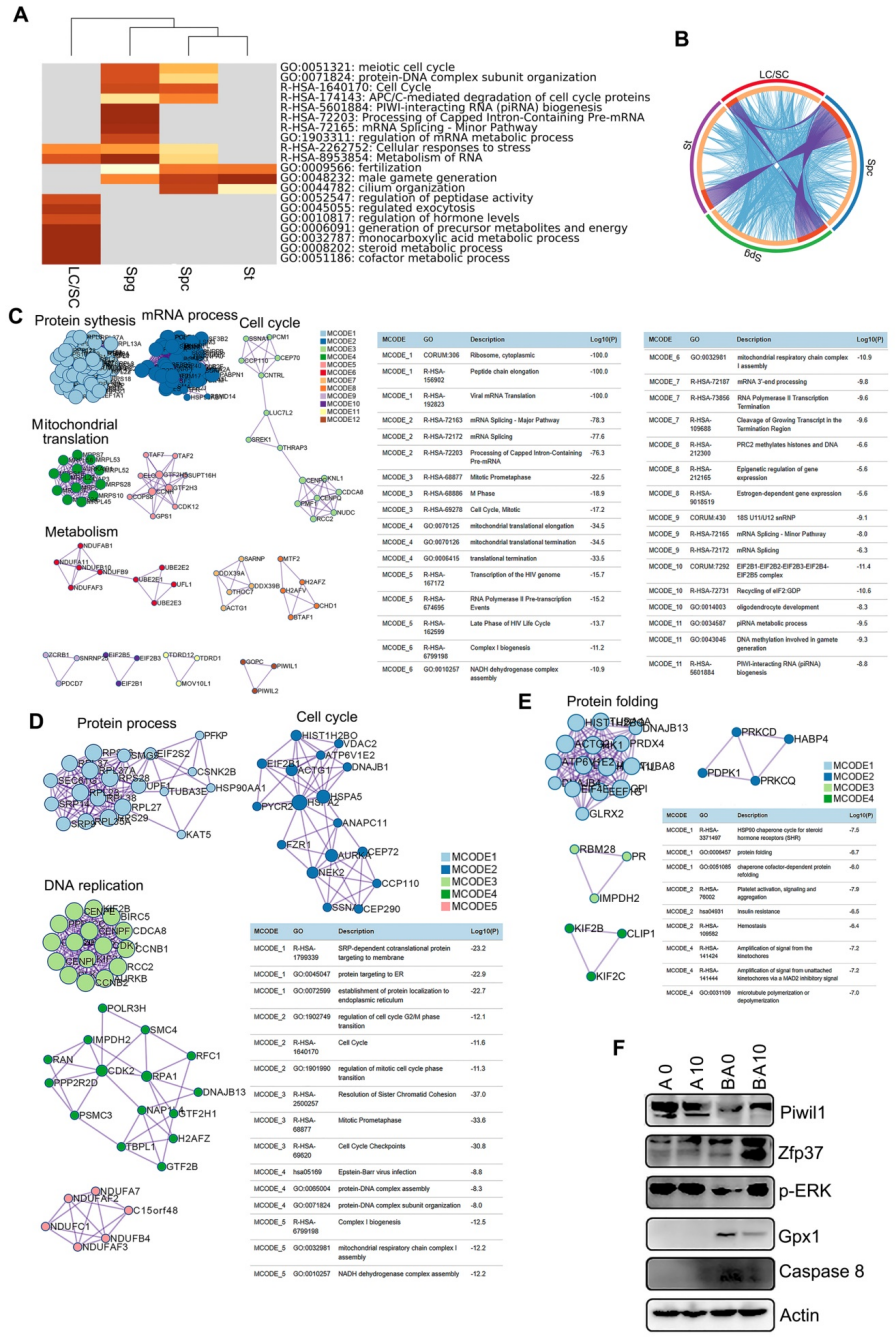
** Enrichment analysis and protein-protein interaction networks for scRNA-seq data.** (**A**) Enrichment analysis for SPGs, SPCs, STs, and LCs/SCs using the online tool in Metascape. (**B**) Circos plots showing interaction between these clusters of cells. The shared marker genes are linked by purple lines, and similar terms are linked by blue lines. (**C**) Protein-protein interaction networks of marker genes in the SPG cluster. (**D**) Protein-protein interaction networks of marker genes in the SPC cluster. (**E**) Protein-protein interaction networks of marker genes in the ST cluster. (**F**) WB of some proteins important for spermatogenesis and maintaining cell function in mouse testis samples.

To identify the functions of the marker genes in each cluster of cells (SPGs, SPCs, STs, and LCs/SCs), we performed multiple enrichment analysis (Figure 3A). The most widely expressed marker genes for the SPG (574 genes), SPC (629 genes), ST (497 genes), and LC/SC (446 genes) clusters were analyzed together (Figure 3A). From the heatmap of enriched terms (top 20 terms) across these four clusters (colored according to *p*-values), we found that 11 terms were enriched in germ cells only, including: “meiotic cell cycle”, “protein-DNA complex subunit organization”, “cell cycle”, “APC/C-mediated degradation of cell cycle proteins”, “PIWI-interacting RNA (piRNA) biogenesis”, and others. Meanwhile, seven terms: “regulation of hormone levels”, “monocarboxylic acid metabolic process”, “steroid metabolic process”, “cofactor metabolic process” and others were enriched in the LC/SC cluster. Furthermore, two terms “cellular responses to stress” and “metabolism of RNA” were enriched in both germ cell (SPG, SPC, ST) and LC/SC clusters (Figure 3A). The data here suggested that marker genes and functions were specific for these clusters. The overlay genes and GO terms are shown in Figure 3B. Most of the overlay terms were in the three germ cell clusters. The first 20 enriched terms for each cluster are presented in Figure S4A-D. The data show the specific functions enriched in each cluster.

Next, we performed protein-protein interaction enrichment analysis for each of the germ cell clusters using marker genes (Figure 3C-E). The networks for each cluster contained the subset of proteins which formed physical interactions with at least one other member in the marker gene set. If the network included 3 to 500 proteins, the Molecular Complex Detection (MCODE) algorithm was used to determine the densely connected network components. For the SPG cluster, there were 12 MCODE and many proteins were connected together for MCODE 1, MCODE 2, MCODE 3, MCODE 4, MCODE 5, MCODE 6, and MCODE 7. Most of the functions in these MCODE were for RNA processes in preparation for meiosis (Figure 3C). There were 5 MCODE for the SPC cluster and many proteins were in each MCODE (Figure 3D). Most of the functions in these MCODE were for cell cycle and protein DNA interactions which are important for spermatogenesis (Figure 3D). There were four MCODE for the ST cluster (Figure 3E).

There were many proteins in MCODE 1, and only three or four proteins for other MCODE. Most of the functions in MCODE 1 involved protein folding, a process that is important in ST maturation (Figure 3E). Moreover, some of the proteins important for spermatogenesis such as piwil1 and ZFP37 were determined by Western blotting. B0 had a decreased protein level of piwil1 compared to AOS 0, while it was increased in the BA10 group (Fig. 3F). Meanwhile, ZFP37 was increased in BA10 (Figure 3F). ERK is important for cell growth and is also involved in the activation of transcriptional factors, we found p-ERK was decreased in B0, while it was increased in BA10 (Figure 3F). Moreover, Gpx1 and caspase 8 were elevated by busulfan alone (B+A 0), while they were reduced in the BA10 group (Figure 3F). The data here indicated that busulfan disrupted spermatogenesis, and that this could be rescued by AOS which suggests that AOS can protect germ cell development from spermatogonia through to spermatocytes and on to spermatids.

### AOS Promoted the Expression of Important Genes for Spermatogenesis *ex vivo*

**Figure 4:**
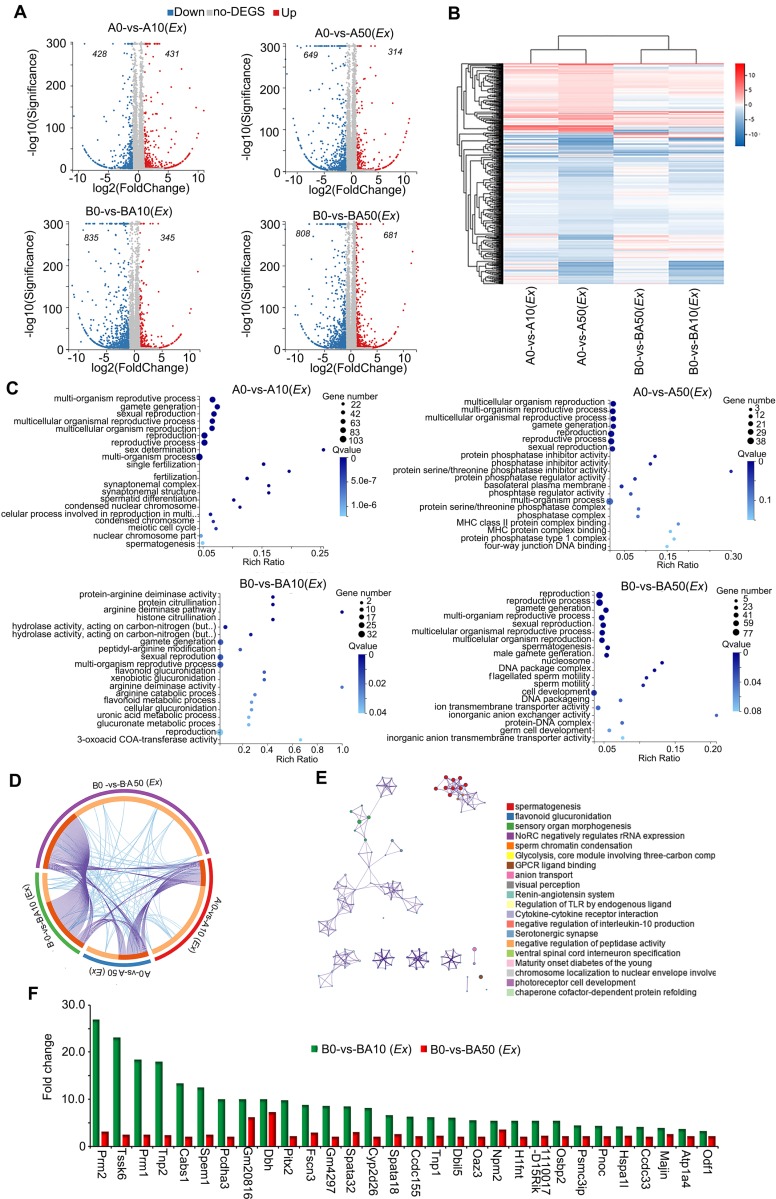
** RNA-seq data for *ex vivo* experiments.** (**A**) Volcano map summary of RNA-seq data in ex v*ivo* experiments. The four comparisons: AOS 0 vs. AOS 10 (*ex vivo*); AOS 0 vs. AOS 50 (*ex vivo*); B+A 0 vs. B+A 10 (*ex vivo*); and B+A 0 vs. B+A 50 (*ex vivo*). (**B**) Heatmap summary of the differentially expressed genes in the four comparisons in the *ex vivo* experiment. (**C**) GO enrichment of up-regulated genes in the four comparisons in the *ex vivo* experiment. (**D**) Circos plots showing interactions between the four comparisons in multiple enrichment analysis in the *ex vivo* experiment. (**E**) Enrichment network of shared marker genes in the comparisons in the *ex vivo* experiment. Each term is indicated by a circular node that is colored according to comparison; nodes that share the same cluster ID are typically close to each other. (**F**) Gene expression comparison of RNA-seq data in the *ex vivo* experiments and the 10x scRNA-seq data in the* in vivo* experiments.

In order to further confirm our *in vivo* data and search for the mode of action of AOS in rescuing spermatogenesis locally in the testis, we performed *ex vivo* experiments. Similar to the *in vivo* experiments, three-week-old male ICR mice were treated with busulfan (40 μg/g body weight) once and subsequently raised normally. Two weeks after busulfan treatment, the testes were collected and cultured with or without AOS (two concentrations 50 μg/mL and 10 μg/mL in culture medium). At the same time, the testes from age-matched mice (no busulfan treatment) were also collected and cultured with or without AOS (two concentrations 50 μg/mL and 10 μg/mL) for 48 h. In all, there were six treatment groups: A0 (*Ex*), A10 (*Ex*), A50 (*Ex*), B0 (*Ex*), BA10 (*Ex*), and BA50 (*Ex*). Testicular tissues were collected for RNA-seq analysis. A data summary is presented in Figure 4A. Compared to A0 (*Ex*), there were 431 genes upregulated and 428 genes down-regulated in A10 (*Ex*). Compared to A0 (*Ex*), 314 genes were increased and 649 genes were decreased in A50 (*Ex*). There were 345 genes up-regulated and 835 genes down-regulated in BA10 (*Ex*) compared to B0 (*Ex*). Compared to B0 (*Ex*), 681 genes were up-regulated and 808 genes were down-regulated in BA50 (*Ex*). The expression levels of the differentially expressed genes in these four comparations [A0 vs. A10 (*Ex*), A0 vs. A50 (*Ex*), B0 vs. BA10 (*Ex*), B0 vs. BA50 (*Ex*)] were analyzed and are presented in Figure 4B. A0 vs. A10 (*Ex*) and A0 vs. A50 (*Ex*) were clustered together, and B0 vs. B10 (*Ex*) and B0 vs. BA50 (*Ex*) were clustered together which suggested that both busulfan and AOS played a major role in the expression of these genes. The up-regulated genes in these four comparisons were analyzed using GO analysis. The genes in each comparison were enriched in reproduction and spermatogenesis related functional pathways (Figure 4C). However, the down-regulated genes in each comparison were enriched in other functional pathways (Figure S5A-D). Moreover, the up-regulated genes in each comparison were analyzed together using multiple enrichment analysis. Many genes and GO terms overlapped in the four comparisons (Figure 4D), which indicated that AOS may play an important role in spermatogenesis. Furthermore, the enriched terms interacted together to form networks (Figure 4E). In these interacted networks, “spermatogenesis” was the most significantly enriched of the four comparisons, which indicated that AOS had a positive effect on spermatogenesis in the *ex vivo* study. Expression of the enriched genes related to spermatogenesis were compared with 10x scRNA-seq data (Figure 4F). In this figure, the green columns denote scRNA-seq data (*in vivo*) and the red columns denote RNA-seq data (*ex vivo*). These genes, including PRM1, PRM2, TNP1, TNP2, and ODF1 are critical during spermatogenesis. Moreover, the data for *in vivo* and *ex vivo* experiments showed the same trends but with lower expression levels in RNA-seq data (*ex vivo*) compared to scRNA-seq data (*in vivo*); this may be because the *ex vivo* study lasted only 48 h while the *in vivo* study lasted five weeks. The data in this section suggested that AOS played an important local role in spermatogenesis.

### AOS Recovered Testicular Metabolites

**Figure 5:**
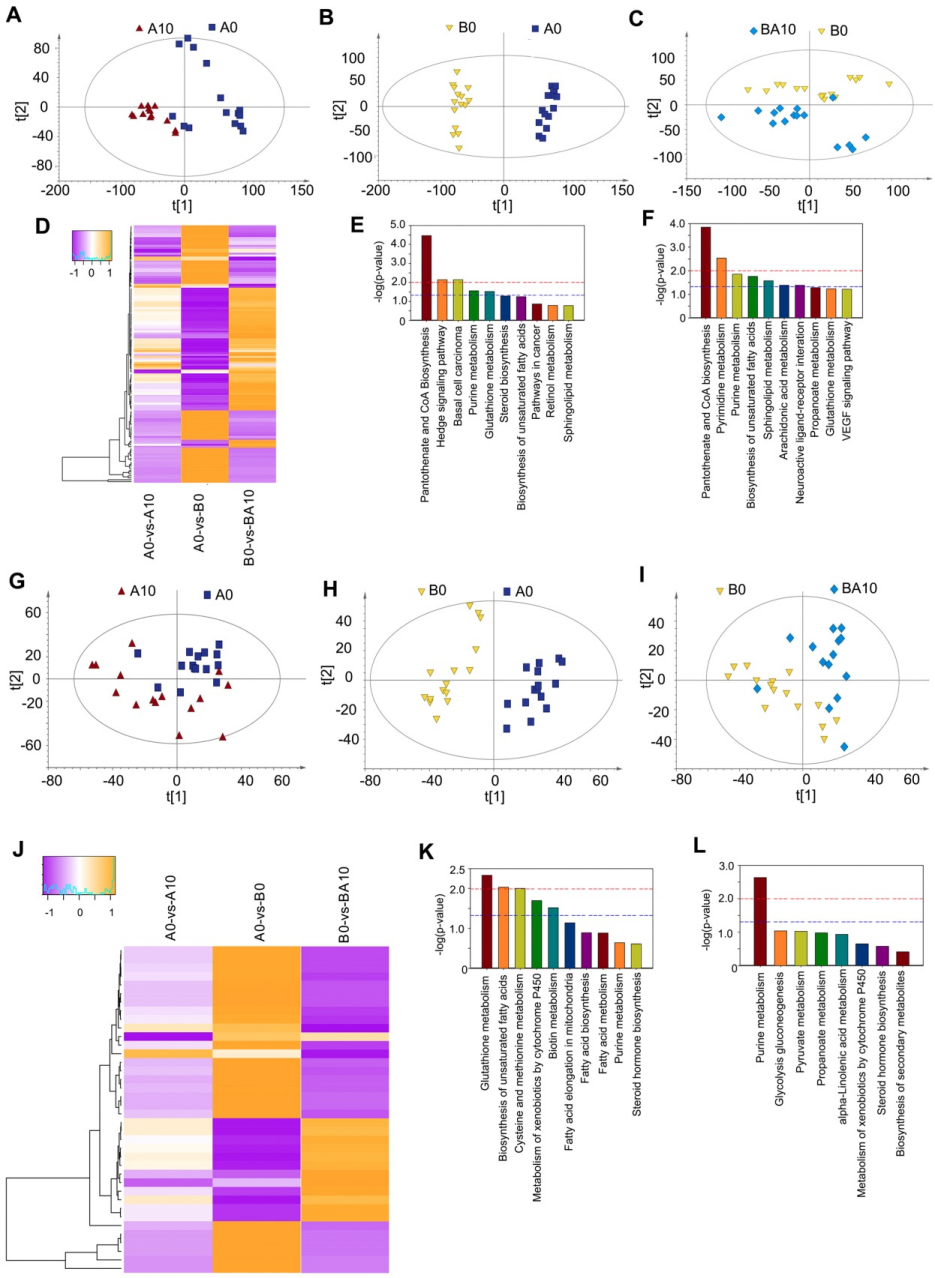
** Plasma and testis metabolome changes.** (**A**) PCA of mouse testis metabolites in the AOS 0 and AOS 10 groups. (**B**) PCA of mouse testis metabolites in the AOS 0 and B+A 0 groups. (**C**) PCA of mouse testis metabolites in the B+A 0 and B+A 10 groups. (**D**) Heatmap of changed testis metabolites. (**E**) Enriched pathways of changed testis metabolites in AOS 0 vs. AOS 10. (**F**) Enriched pathways of changed testis metabolites in B+A 0 vs. B+A 10. (**G**) PCA of mouse plasma metabolites in the AOS 0 and AOS 10 groups. (**H**) PCA of mouse plasma metabolites in the AOS 0 and B+A 0 groups. (**I**) PCA of mouse plasma metabolites in the B+A 0 and B+A 10 groups. (**J**) Heatmap of changed plasma metabolites. (**K**) Enriched pathways of changed plasma metabolites in AOS 0 vs. AOS 10. (**L**) Enriched pathways of changed plasma metabolites in B+A 0 vs. B+A 10.

After finding that AOS could rescue spermatogenesis in the testis, next we set out to explore the role of AOS in rescuing the testicular microenvironment. Testicular metabolites (*in vivo* study) were determined by UPLC-Q-TOF/MS. Data were analyzed by PCoA analysis, and PCoA score plots showed that the groups in the following pairings could be clearly separated: A10 and A0 (Figure 5A), B0 and A0 (Figure 5B), and BA10 and B0 (Figure 5C). The data suggested that both AOS and busulfan influenced metabolic profiles in mouse testes. There were 313, 428, and 330 significantly changed metabolites (positive and negative modes together) for the following three comparisons: A0 vs. A10, A0 vs. B0, and B0 vs. BA10, respectively (Table S5). One hundred and thirty-two compounds were common between these three comparisons. The expression of these 132 compounds was very interesting because 65 compounds were increased by busulfan (in A0 vs. B0) while they were decreased by AOS (in B0 vs. BA10), and 67 compounds were decreased by busulfan (in A0 vs. B0), however, they were increased by AOS (in B0 vs. BA10; Figure 5D). The data indicated that AOS rescued the compounds that were disturbed by busulfan. It was even more interesting that glutathione and its precursor gamma-glutamylcysteine were increased by AOS.

Glutathione is an excellent antioxidant which plays a vital role in protecting biological systems 32. Glutathione was increased 1.69- and 2.25-fold in A0 vs. A10 and B0 vs. BA10, respectively, while it was decreased by busulfan to 0.23-fold in A0 vs. B0 (Table S5). Gamma-glutamylcysteine was increased 1.82- and 2.44-fold in A0 vs. A10 and B0 vs. BA10, respectively, while it was decreased by busulfan to 0.25-fold in A0 vs. B0 (Table S5). Furthermore, many lipids or phospholipids were altered by these treatments.

The function of these differentially expressed compounds were analyzed using the KEGG database and the most enriched pathways in A0 vs. A10 were: pantothenate and CoA biosynthesis, hedgehog signaling pathway, purine metabolism, glutathione metabolism, steroid biosynthesis, and the biosynthesis of unsaturated fatty acids (Figure 5E). The most enriched pathways in B0 vs. BA10 were: pantothenate and CoA biosynthesis, pyrimidine metabolism, purine metabolism, biosynthesis of unsaturated fatty acids, sphingolipid metabolism, arachidonic acid metabolism, neuronactive ligand-receptor interaction, propanoate metabolism, and glutathione metabolism (Figure 5F). Metabolism plays a critical role in spermatogenesis 33, and lipid metabolism is also known to be important in spermatogenesis and male fertility 34,35.

### AOS Improved Blood Metabolites

Since AOS can improve the testicular microenvironment, next we investigated whether AOS could affect blood metabolism and the correlation between blood metabolism and testis metabolism. The PCoA score plots revealed that the groups in the following pairings could be clearly separated: A10 and A0 (Figure 5G), B0 and A0 (Figure 5H), and BA10 and B0 (Figure 5I). Data indicated that both AOS and busulfan changed the metabolic profiles in blood. There were 105, 137, and 76 significantly altered metabolites (positive and negative modes together) for the following three blood sample comparisons: A0 vs. A10, A0 vs. B0, and B0 vs. BA10, respectively (Table S6). Thirty-eight compounds were common to all three comparisons. The expression of these 38 compounds was also very interesting because 26 compounds were increased by busulfan (in A0 vs. B0) while they were reduced by AOS (in B0 vs. BA10), and 12 compounds were reduced by busulfan (in A0 vs. B0), however they were increased by AOS (in B0 vs. BA10; Figure 5J). The data indicated that AOS rescued the compounds that were disturbed by busulfan in mouse blood.

The functions of these differentially expressed compounds were analyzed using the KEGG database. The most enriched pathways in A0 vs. A10 are presented in Figure 5K, including glutathione metabolism, biosynthesis of unsaturated fatty acids, fatty acid elongation in mitochondria, fatty acid biosynthesis, fatty acid metabolism, pyrimidine metabolism, and others. The most enriched pathways in B0 vs. BA10 were alpha-linolenic acid metabolism, pyrimidine metabolism, steroid hormone biosynthesis, and others (Figure 5L). The data indicated that the most changed metabolites were related to lipid metabolism, a process that is very important for spermatogenesis and male fertility 34,35. The correlation of blood metabolites and testis metabolite was determined based in Spearman's correlation coefficient. Most of the metabolites were positively correlated together (Table S7). Moreover, the data suggested that AOS might improve small intestine function and the microbiota in the small intestine through assisting digestion and absorption.

### AOS Improved Intestinal Microbiota

**Figure 6:**
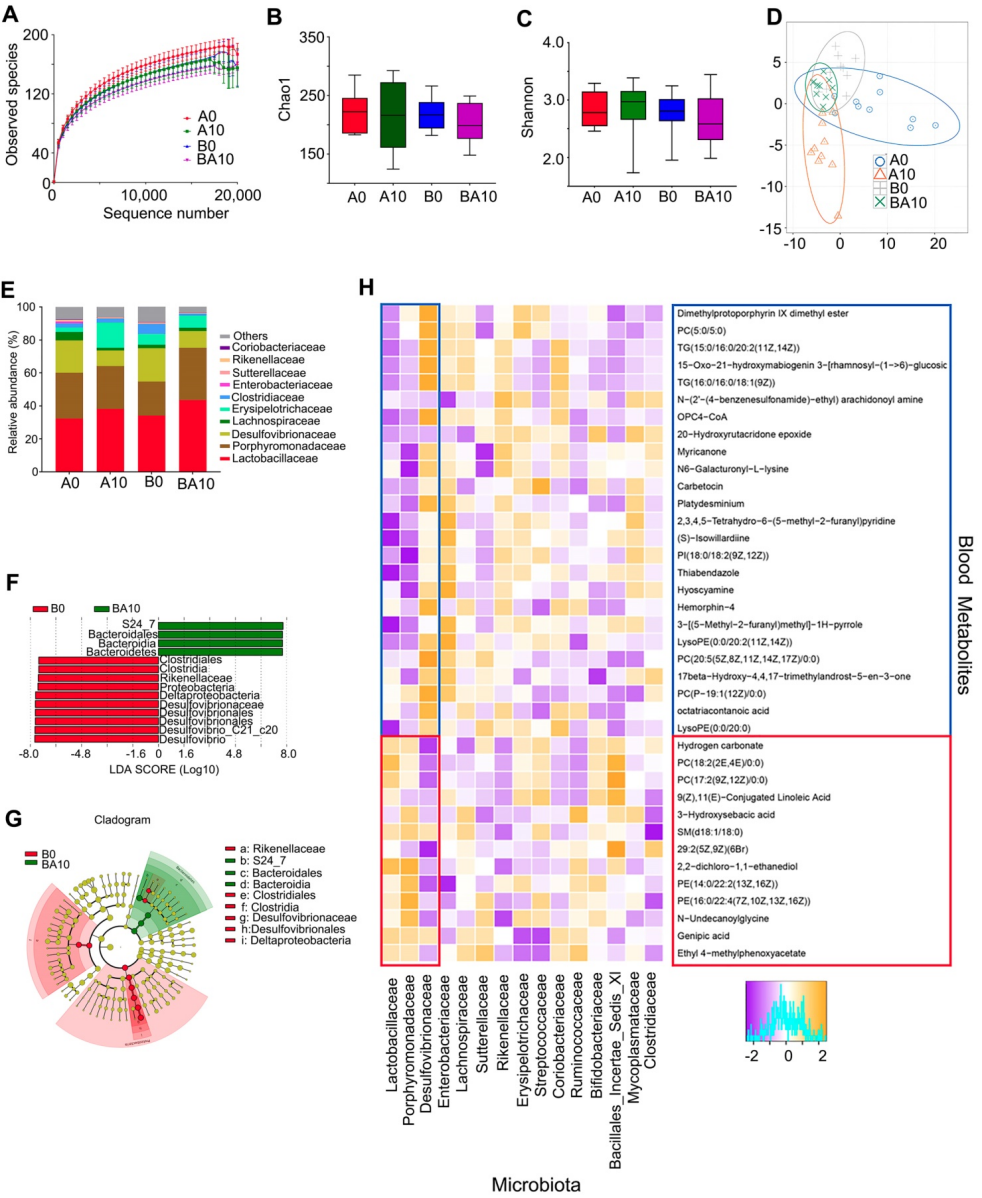
** Small intestinal microbiota changes and correlation of changed intestinal microbiota and changed plasma metabolites.** The alpha index of the small intestine microbiota: (**A**) Rarefaction curve; (**B**) Chao1 index; (**C**) Shannon index. (**D**) The PLS-DA of the microflora in different treatments. (**E**) Differences of bacterial abundance at the family level. (**F**) LDA distribution. (**G**) Cladogram. Linear discriminate analysis effect size (LEfSe) was performed to determine the difference in abundance; the threshold of LDA score was 4.0. (n = 10 samples/group)

In order to examine whether the rescuing effect of AOS on spermatogenesis was associated with gut microbiota, the bacterial 16s rRNA V3-4 region of intestinal digesta was sequenced. The rarefaction curve revealed that the data were reliable for further analysis (Figure 6A). There was almost no difference between the four treatment groups in richness (Chao1) and diversity (Shannon) based on the alpha diversity index (Figure 6B-C). Moreover, the OUT based PLS-DA analysis showed that these four treatment groups could be easily separated, especially the A0 and A10 groups (Figure 6D). AOS altered the relative abundance of the predominant bacteria in murine intestinal digesta (Figure 6E; Table S8). The three predominant bacteria were Lactobacillaceae, Porphyromonadaceae, and Desulfovibrionaceae, while the three relatively less-predominant bacteria were Lachnospiraceae, Erysipelotrichaceae, and Clostridiaceae (Figure 6E). AOS increased the percentage of Lactobacillaceae from 32.21% (in A0) to 38.12% (A10), and from 34.03% (in B0) to 43.60% (BA10; Table S8). However, AOS decreased the percentage of Desulfovibrionaceae from 19.57% (in A0) to 9.41% (A10), and from 20.20% (in B0) to 10.17% (BA10; Table S8). AOS had little effect on the other predominant bacteria in intestinal digesta. Moreover, linear discriminant effect size (LEfSe) analysis revealed that Proteobacteria (a phylum of Desulfovibrionaceae) were significantly enriched in the B0 group, but not in the BA10 group (Figure 6F-G). However, Bacteroidales was enriched in BA10 but not in B0 (Figure 6F-G). The data suggested that AOS can increase “beneficial” bacteria such as Bacteroidales and Lactobacillaceae and it can also decrease “harmful” bacteria, such as Desulfovibrionaceae 36-38 to improve the intestinal microenvironment.

Metabolomics is an excellent tool for investigating crosstalk between the host and gut microbiota 39. Therefore, Spearman's correlation coefficient was calculated between plasma metabolites and gut microbiota. As shown in Figure 6H, fifteen microflora families, including Lactobacillaceae, Porphyromonadaceae, Desulfovibrionaceae, Enterobacteriaceae, Lachnospiraceae, Sutterellaceae, Rikenellaceae, Erysipelotrichaceae, Streptococcaceae, Coriobacteriaceae, Ruminococcaceae, Bifidobacteriaceae, Bacillales_Incertae_Sedis_XI, Mycoplasmataceae, and Clostridiaceae had correlations with the 38 metabolites (common plasma metabolites in the following three comparisons: A0 vs. A10, A0 vs. B0, and B0 vs. BA10; Figure 6H).

Lactobacillaceae and Desulfovibrionaceae were best correlated with these 38 metabolites. The 26 compounds that were increased by busulfan (in A0 vs. B0) and decreased by AOS (in B0 vs. BA10) were negatively correlated with Lactobacillaceae. Lactobacillaceae were more abundant in AOS treatment groups (in A10, and BA10) and less so in A0 and B0. The 12 compounds that were decreased by busulfan (in A0 vs. B0) and increased by AOS (in B0 vs. BA10) were positively correlated with Lactobacillaceae. The correlation of Desulfovibrionaceae and blood metabolites was opposite to that between Lactobacillaceae and blood metabolites. Desulfovibrionaceae was more abundant in non-AOS treatment groups (A0, and B0) and it was positively correlated with the 26 compounds that were increased by busulfan (in A0 vs. B0) and decreased by AOS (in B0 vs. BA10). However, Desulfovibrionaceae was less abundant in AOS treatment groups (A10, and BA10), and it was negatively correlated with the 12 compounds that were decreased by busulfan (in A0 vs. B0) and increased by AOS (in B0 vs. BA10). The data further indicated that these two dominant bacteria may be involved in AOS modification of blood metabolites which may assist in the rescue of spermatogenesis.

## Discussion

Worldwide, the incidence of cancer is continuing to increase 8-10 and one of the most prevalent cancers during reproductive age is leukemia 40,41. Chemotherapy with alkylating agents such as busulfan is an effective management for leukemia especially in children; however, busulfan adversely affects the male reproductive system, resulting in oligospermia or azoospermia, and finally permanent male sterility 8,15,16,42. Moreover, it has been reported that busulfan-induced male sterility in mice is very similar to that in humans 43,44. In the current investigation, we found that one dose of busulfan (40 mg/Kg BW at 3 weeks of age) produced borderline azoospermia in mice during adulthood (8 weeks of age) which is consistent with many previous studies 5-7. However, busulfan plus AOS treatment (BA10) increased sperm concentration and motility more than three-fold compared to busulfan alone, which suggested that AOS can rescue spermatogenesis. Therefore, we set out to explore the underlying mechanisms by which AOS improves spermatogenesis by using 10x single cell RNA sequencing analysis (scRNA-seq). In corroboration with recent studies using scRNA-seq on human testis samples, we found similar cell types in mouse testis samples 1,2. Germ cells can be separated into three major clusters: SPGs, SPCs, and STs; SCs and LCs can also be clustered together. AOS alone (A10) had little effect on the proportion of germ cells compared to the control (A0). Busulfan (B0) drastically decreased the proportion of SPCs and STs, with the most common cellular type in these mice being SCs and LCs. Busulfan plus AOS (BA10) significantly increased the proportion of SPGs and SPCs compared to B0. The percentage of STs was also higher in BA10 compared to B0, however, it was lower than that in A0 and A10. Based on our data, we proposed that AOS protected germ cell development in testes that had undergone busulfan treatment. Moreover, the beneficial effects of AOS on male germ cell development were mainly due to the recovery of gene expression because AOS was able to increase the expression and protein levels of the prominent genes affecting spermatogenesis. These beneficial effects may be the direct consequence of AOS on testicular germ cells as the *ex vivo* (testis culture) and *in vivo* data were consistent. Under these two models, AOS was able to increase the expression of genes important to spermatogenesis. Deeper mechanisms may involve AOS in the regulation of transcriptional factors which are important in controlling gene expression.

It is known that metabolic regulation is essential for spermatogenesis 33,45,46, and cholesterol and lipid homeostasis play a vital role in male fecundity 47-51. Acting as nurse cells, Sertoli cells provide the nutrients and energy for germ cell development. Many components such as hormones and other endogenous or exogenous factors have a synergistic contribution to the homeostasis of metabolism in the testis and the progression of spermatogenesis 33. It is known that the abnormal metabolism of lipids in the reproductive system or blood contributes to male infertility in humans 49-51. In this study, we found that busulfan upset the homeostasis of lipid metabolism in murine blood and testis samples, while AOS reversed this change. These findings suggested that AOS can regulate metabolism, especially lipid homeostasis, to improve sperm development; indeed, this is the first recorded finding of AOS regulating metabolomes in the blood and testes.

Recently, there has been a rising interest regarding the effect of the gut microbiome on human physiology. It not only plays roles in metabolic related disorders such as obesity and diabetes 52,53, but it also affects other systems such as the nervous system and reproductive system 54-56. It has been reported that the gut microbiota can influence reproductive performance in both males and females as well as their offspring 56. In this study, we found that AOS increased the “beneficial” bacteria such as Bacteroidales and Lactobacillaceae while it decreased “harmful” bacteria in murine small intestines. Gut microbiota can metabolize nutrients in the intestine and can also regulate intestinal metabolites to influence the blood metabolome 52,53. In turn, while travelling through other organs, blood metabolites can influence their development or cause disorders 54-56. Our current study suggested that the blood and testis metabolome and gut microbiota interacted together under AOS treatment to mitigate busulfan disruption of spermatogenesis.

AOS rescued busulfan disrupted spermatogenesis by improving germ cell development, the testis and blood metabolome, and gut microbiota. These beneficial advantages of AOS can be used to improve male reproduction in patients under busulfan or other cancer-drug treatments. Worldwide, 20%-35% of couples are infertile 1,3,57, and many of them have idiopathic failed gametogenesis (spermatogenesis); we propose that AOS may have implications for these infertile couples through the improvement of spermatogenesis.

## Materials and Methods

### Study design: *In vivo* and *ex vivo*

All animal procedures were approved and conducted in accordance with the Qingdao Agriculture University Animal Care and Use Committee. Mice were maintained under a light:dark cycle of 12:12 h, at a temperature of 23 ℃ and humidity of 50%-70%; they had free access to food (chow diet) and water 58.

### *In vivo*: Mouse exposure to busulfan and/or AOS

Three-week-old ICR male mice were given a single injection of busulfan (40 mg/kg BW) 5. The following day, the mice were dosed with ddH_2_O as the control or AOS 10 mg/kg BW via oral gavage (0.1 ml/mouse/d). Our preliminary experiments found that 10 mg/kg was the optimum concentration for rescuing murine spermatogenesis disrupted by busulfan. AOS dosing solution was freshly prepared on a daily basis and delivered every morning for two weeks. There were four treatment groups (30 mice/treatment): (1) A0 (vehicle control, ddH_2_O); (2) A10 (AOS 10 mg/kg BW); (3) B0 (busulfan alone); and (4) BA10 (busulfan plus AOS 10 mg/kg BW). After treatment, the mice were humanely euthanized to collect samples for different analyses.

### *Ex vivo*: Mouse testes exposure to busulfan and/or AOS

Three-week-old ICR male mice were given a single injection of 40 mg/kg BW of busulfan 5; the mice were then raised normally. After two weeks, the testes were collected for culture. Meanwhile, similar age matched non-busulfan treated mouse testes were also collected for culture. There were six treatment groups (six testes/treatment; repeated three times): (1) A0 (*ex vivo*) (DMEM/F12 medium with 10% FBS); (2) A10 (*ex vivo*) (AOS 10 μg/mL in DMEM/F12 medium with 10% FBS); (3) A50 (*ex vivo*) (AOS 50 μg/mL in DMEM/F12 medium with 10% FBS); (4) B0 (busulfan alone; DMEM/F12 medium with 10% FBS); (5) BA10 (*ex vivo*) (busulfan plus AOS 10 μg/mL in DMEM/F12 medium with 10% FBS); (6) BA50 (*ex vivo*) (busulfan plus AOS 50 μg/mL in DMEM/F12 medium with 10% FBS). Culture took place in an incubator at 37 ℃ and 5% CO_2_ for 48 h. Subsequently the cultured testes were collected for isolation of total RNA; these samples then underwent RNA-seq analysis.

### Evaluation of spermatozoa motility using a computer-assisted sperm analysis system

Spermatozoa motility was assessed using a computer-assisted sperm assay (CASA) method according to World Health Organization guidelines 57,58.

### Morphological observations of spermatozoa

The resected murine caudal epididymides were placed in RPMI medium, finely chopped, and then Eosin Y (1%) was added for staining as described previously 57,58.

### Assessment of acrosome integrity

Acrosomal integrity was determined by an intense staining on the anterior region of the sperm head under bright-field microscopy (AH3-RFCA, Olympus, Tokyo, Japan) and scored accordingly 57,58.

### Single cell library preparation, sequencing, and data analysis [single cell RNA-sequencing (scRNA-seq)]

***Single cell library preparation and sequencing.***Single cell libraries were constructed with a 10x Genomics Chromium Single Cell 3′ Library & Gel Bead Kit v2 (10× Genomics Inc., Pleasanton, CA, USA, 120237) following the manufacturer's instructions. Single cell sample collection followed the methods reported by Wang et al. 1. Briefly, mouse testes were resected, seminiferous tubules were cut into small pieces, and then washed with PBS three times to remove the spermatozoa. Subsequently, the tissue was digested using TrypLE express (Invitrogen) for 15 min at 37 °C (in culture, in an incubator). The single cells were collected by filtration using a 40 μm filter. Cells were then washed twice with PBS solution supplemented with 0.04% bovine serum albumin (BSA, Sigma, St. Louis, MO, USA, A1933). Trypan blue staining and a hemocytometer (Bio-Rad, Hercules, CA, USA, TC20) were used to detect cell viability. Six individual mouse testicular cells were collected, combined together, then a concentration of 1000 cells/μl was used for loading onto the single cell chip (one/group). A Chromium 10x Single Cell System (10×Genomics) was used to form the Gel-Bead in Emulsions (GEMs). Cells were then barcoded and a cDNA library was constructed. The sequencing protocol used an Illumina HiSeq X Ten sequencer (Illumina, San Diego, CA, USA) with pair end 150 bp (PE150) reads.

***Single sample analysis and aggregation.*** CellRanger v2.2.0 software (https://www.10xgenomics.com/) was used to process the datasets using the “--force-cells = 5000” argument. The 10x Genomics pre-built mouse genome for mm10-3.0.0 (https://support.10xgenomics.com/single-cell-geneexpression/software/downloads/ latest) was referenced 59.

***Subclustering and gene ontology enrichment analysis*.** After characterization of all cell clusters in murine small intestine samples, cells were further clustered based on their cell identity. To obtain the same type of cells for downstream analysis, the “SubsetData” function was applied. After clustering, cluster-specific marker genes were identified using the “FindAllMarkers” function. The marker genes were used for enrichment analysis in Metascape (http://metascape.org).

***Single-cell pseudo-time trajectory analysis*.** Monocle 2 (v2.8.0) was used to determine the single-cell pseudo-time trajectory (http://cole-trapnell-lab.github.io/monocle-release/tutorials/) 60,61. The Monocle object was formed using the Monocle implemented “newCellDataSet” function from the Seurat object with a lowerDetectionLimit = 0.5.

***Single cell regulatory network analysis*.** To find the gene regulatory networks during small intestine cell development, we performed regulatory network inference and clustering using SCENIC (https://github.com/aertslab/SCENIC), a modified method for inferring with gene regulatory networks from single-cell RNA-seq data 31.

### RNA-seq analysis for ex vivo testes samples

Transcriptomics were analyzed as described in our early articles 62.

### Sequencing of microbiota from small intestine digesta samples and data analysis 63

***DNA Extraction*.** Total genomic DNA of small intestine digesta was isolated using an E.Z.N.A.R Stool DNA Kit (Omega Bio-tek Inc., Norcross, GA, USA) following the manufacturer's instructions. DNA quantity and quality were analyzed using NanoDrop 2000 (Thermo Scientific, USA) and 1% agarose gel. Ten samples/groups were determined.

***Library preparation and sequencing.*** The V3-V4 region of the 16S rRNA gene was amplified using the primers MPRK341F (50-ACTCCTACGGGAGGCAGCAG-30) and MPRK806R: (50-GGACTACHVGGGTWTCTAAT -30) with Barcode.

***Analysis of sequencing data.*** Operational taxonomic unit abundance information was normalized using a standard of sequence number corresponding to the sample with the least sequences. The alpha diversity index was calculated with QIIME (Version 1.7.0). The Unifrac distance was obtained using QIIME (v. 1.7.0), and PCoA (principal coordinate analysis) was performed using R software (Version 2.15.3). The linear discriminate analysis effect size (LEfSe) was performed to determine differences in abundance; the threshold LDA score was 4.0. GraphPad Prism7 software was used to produce the graphs.

### Plasma and testis metabolite measurements by LC-MS/MS

Plasma samples were collected and immediately stored at -80 °C. Before LC-MS/MS analysis, the samples were thawed on ice and processed to remove proteins. Testis samples were collected and the same amount of tissue from each mouse testis was used to isolate the metabolites using CH3OH: H2O (V:V) = 4:1. Then samples were detected by ACQUITY UPLC and AB Sciex Triple TOF 5600 (LC/MS) as reported previously 39. Fifteen samples/group were analyzed for plasma or testis samples.

### Histopathological analysis

Testicular tissues were fixed in 10% neutral buffered formalin, paraffin embedded, cut into 5 μm sections, and subsequently stained with hematoxylin and eosin (H&E) for histopathological analysis.

### Immunofluorescence staining (IHF)

The procedure for immunofluorescence staining is reported in our recent publications 58,64. Table S9 lists the primary antibodies.

### Western blotting

Western blotting analysis followed the procedure reported in our previous publications 58,64. Briefly, testis tissue samples were lysed in RIPA buffer containing a protease inhibitor cocktail from Sangong Biotech, Ltd. (Shanghai, China). Protein concentration was determined by BCA kit (Beyotime Institute of Biotechnology, Shanghai, P.R. China). Information for primary antibodies is given in Table S9.

### Statistical analysis

Data were analyzed using SPSS statistical software (IBM Co., NY, USA) with one-way analysis of variance (ANOVA) followed by LSD multiple comparison tests. All groups were compared with each other for every parameter. Data is shown as the mean ± SEM. Statistical significance was based on *p* < 0.05.

## Supplementary Material

Supplementary materials and methods, figures, and tables.
