# Supplementary material for: Alginate oligosaccharides improve germ cell development and testicular microenvironment to rescue busulfan disrupted spermatogenesis
Source: Theranostics. 2020 Feb 10;10(7):3308–24. doi: 10.7150/thno.43189 (PMC7053202; doi:10.7150/thno.43189)
Supplement: Supplementary file 1 — Supplementary materials and methods, figures, and tables. [file thnov10p3308s1.pdf]

Supplemental Information

Detailed Materials and Methods

Evaluation of spermatozoa motility using a computer-assisted sperm analysis system

Spermatozoa motility was assessed using a computer-assisted sperm assay (CASA) method according to World Health Organization guidelines [57,58]. After euthanasia, spermatozoa were collected from the cauda epididymis of mice and suspended in DMEM/F12 medium with 10% FBS and incubated at 37.5 °C for 30 min; samples were then placed in a pre-warmed counting chamber [57,58]. A microscopic sperm class analyzer (CASA; MICROPTIC S.L. Barcelona, Spain) was used in this investigation. It was equipped with a 20-fold objective, a camera adaptor (Eclipse E200, Nikon, Japan), and a camera (acA780-75gc, Basler, Germany). The classification of sperm motility was as follows: Grade A linear velocity >22 μm s^{-1}; Grade B <22 μm s^{-1} and curvilinear velocity >5 μm s^{-1}; Grade C curvilinear velocity <5 μm s^{-1}; and Grade D = immotile spermatozoa. The spermatozoa motility data represented only Grade A + Grade B since only these two grades are considered to be functional.

Morphological observations of spermatozoa

*T*he extracted murine caudal epididymides were placed in RPMI medium and finely chopped; subsequently, Eosin Y (1%) was added for staining as described previously [57,58]. Spermatozoon abnormalities were then viewed using an optical microscope and were classified into head or tail morphological abnormalities: two heads, two tails, blunt hooks, and short tails. The examinations were repeated three times, and 500 spermatozoa per animal were scored.

Assessment of acrosome integrity

After harvest, mouse spermatozoa were incubated at 37.5 °C for 30 min, after

which a drop of sperm suspension was uniformly smeared on a clean glass slide. Smeared slides were air dried and incubated in methanol for 2 min for fixation. After fixation, the slides were washed with PBS three times. Assessment of an intact acrosome was accomplished by staining the sperm with 0.025% Coomassie brilliant blue G-250 in 40% methanol for 20 min at room temperature (RT). The slides were then washed three times with PBS and mounted with 50% glycerol in PBS. Acrosomal integrity was determined by an intense staining on the anterior region of the sperm head under bright-field microscopy (AH3-RFCA, Olympus, Tokyo, Japan) and scored accordingly [57,58].

Single cell library preparation, sequencing, and data analysis [single cell RNA-sequencing (scRNA-seq)]

Single cell library preparation and sequencing. Single cell libraries were constructed with the 10x Genomics Chromium Single Cell 3′ Library and Gel Bead Kit v2 (10× Genomics Inc., Pleasanton, CA, USA, 120237) following the manufacturer's instructions. Single cell sample collection followed the methods reported by Wang et al. [1]. Briefly, mouse testes were collected, seminiferous tubules were cut into small pieces, and then washed with PBS three times to remove the sperm. Subsequently, the tissue was digested using TrypLE express (Invitrogen) for 15 min at 37 °C (in culture, in an incubator). The single cells were collected by filtration using a 40 μm filter. Cells were then washed twice with PBS solution supplemented with 0.04% bovine serum albumin (BSA; Sigma, St. Louis, MO, USA, A1933). Trypan blue staining with a hemocytometer (Bio-Rad, Hercules, CA, USA, TC20) was used to detect cell viability. Six individual murine testicular cells were collected and combined together; subsequently, a concentration of 1000 cells/μl was used for loading onto the single cell chip (one/group). A Chromium 10x Single Cell System (10×Genomics, Pleasanton, CA,

USA) was used to form the Gel-Bead in Emulsions (GEMs). Cells were then barcoded and a cDNA library was constructed. The sequencing protocol used an Illumina HiSeq X Ten sequencer (Illumina, San Diego, CA, USA) with pair end 150 bp (PE150) reads.

Single sample analysis and aggregation. CellRanger v2.2.0 software (https://www.10xgenomics.com/) was used to process the datasets with the "--force-cells = 5000" argument. The 10x Genomics pre-built mouse genome for mm10-3.0.0 (https://support.10xgenomics.com/single-cell-geneexpression/software/downloads/latest) was referenced. After the CellRanger analysis, the gene-barcode matrices were processed using the Seurat single cell RNA-seq analysis R package in Rstudio (v3.0) [59]. Cells with minimal genes <200 and genes expressed in <3 cells were removed to obtain high-quality datasets for downstream analysis. After normalization, the three datasets (from the three treatment groups) were merged together using the Seurat "RunMultiCCA" function. The characterized cell clusters were reviewed by the Seurat "RunTSNE" function based on the t-distributed Stochastic Neighbor Embedding (tSNE) algorithm with default settings. The cell clusters were calculated using the "FindClusters" function. The Seurat "FindAllMarkers" function was applied to find cell cluster markers.

Subclustering and gene ontology enrichment analysis. After characterization of all cell clusters in murine small intestine samples, cells were further clustered based on their cell identity. To obtain the same type of cells for downstream analysis, the "SubsetData" function was applied. After clustering, cluster-specific marker genes were identified using the "FindAllMarkers" function. The marker genes were used for enrichment analysis in Metascape (http://metascape.org).

Single-cell pseudo-time trajectory analysis. Monocle 2 (v2.8.0) was used to determine the single-cell pseudo-time trajectory (http://cole-trapnell-

lab.github.io/monocle-release/tutorials/) [60,61]. The Monocle object was formed by implementing the Monocle "newCellDataSet" function from the Seurat object with a "lowerDetectionLimit" = 0.5. The variable genes for ordering were obtained using Seurat. Dimensionality was constructed using the DDRTree method with regression of the number of UMIs. Root states were collected following their Seurat cell identity information and branch-specific gene expression was calculated using the Monocle implemented BEAM function. The branched heatmap was further constructed using the "plot_genes_branched_heatmap" function.

Single cell regulatory network analysis. To identify gene regulatory networks active during the development of the small intestine, we performed regulatory network inference and clustering using SCENIC (https://github.com/aertslab/SCENIC), a modified method for inferring with gene regulatory networks from single-cell RNA-seq data [31]. The single-cell RNA-seq expression matrix, where each column represents a cell ID and each row represents a gene, was used for analysis. Then, the "geneFiltering" function was used to remove genes with UMI counts across all samples <100 and was expressed in less than 1% of cells. GENIE3 was then used to infer the co-expression matrix containing potential regulators. To identify potential direct-binding targets, RcisTarget was used based on DNA-motif analysis and we used databases (mm 10) that scored the motifs in the gene promoters (up to 500 bp upstream the TSS), and in the 10 kb around the TSS (+/-10 kb). The AUCell algorithm was used to calculate regulon activity in each cell and to convert the network activity into ON/OFF (binary activity matrix) with default settings.

RNA-seq analysis for ex vivo testes samples

Transcriptomics were analyzed as described in our earlier article [62]. Briefly, total RNA was isolated using TRIzol Reagent (Invitrogen) and purified using a Pure-

Link1 RNA Mini Kit (Cat: 12183018A; Life Technologies) following the manufacturer's protocol. The total RNA samples were first treated with DNase I to degrade any possible DNA contamination. Then the mRNA was enriched using oligo(dT) magnetic beads. When mixed with the fragmentation buffer, the mRNA was broken into short fragments (about 200 bp), after which the first strand of cDNA was synthesized using a random hexamer-primer. Buffer, dNTPs, RNase H, and DNA polymerase I were added to synthesize the second strand. The double strand cDNA was purified with magnetic beads. Subsequently, 3'-end single nucleotide A (adenine) addition was performed. Finally, sequencing adaptors were ligated to the fragments. The fragments were enriched by PCR amplification. During the QC step, Agilent 2100 Bioanalyzer and ABI StepOnePlus Real-Time PCR System were used to qualify and quantify the sample library. The library products were ready for sequencing via Illumina HiSeqTM 2500. The reads were mapped to reference genes using SOAPaligner (v. 2.20) with a maximum of two nucleotide mismatches allowed at the parameters of "-m 0 -x 1000 -s 40 -l 35 -v 3 -r 2". The read number of each gene was transformed into RPKM (Reads Per Kilo bases per Million reads), and then differentially expressed genes were identified using the DEGseq package and the MARS (MA-plot-based method with the Random Sampling model) method. The threshold was set as FDR $\leq$0.001 and an absolute value of $\log_2$ ratio $\geq$1 to judge the significance of differences in gene expression.

Sequencing of microbiota from small intestine digesta samples and data analysis [63]

DNA Extraction. Total genomic DNA of small intestine digesta was isolated using an E.Z.N.A.R Stool DNA Kit (Omega Bio-tek Inc., USA) following the manufacturer's instructions. DNA quantity and quality were analyzed using a NanoDrop 2000 (Thermo Scientific) and 1% agarose gel. Ten samples/groups were determined.

Library preparation and sequencing. The V3–V4 region of the 16S rRNA gene was amplified using the primers MPRK341F (50- ACTCCTACGGGAGGCAGCAG -30) and MPRK806R: (50- GGACTACHVGGGTWTCTAAT -30) with Barcode. The PCR reactions (total 30 μL) included 15 μL of PhusionR High-Fidelity PCR Master Mix (New England Biolabs), 0.2 mM of primers, and 10 ng of DNA. The thermal cycle was carried out with an initial denaturation at 98 °C, followed by 30 cycles of 98 °C for 10 s, 50 °C for 30 s, 72 °C for 30 s, and a final extension at 72 °C for 5 min. PCR products were purified using a GeneJET Gel Extraction Kit (Thermo Scientific). The sequencing libraries were constructed with a NEBNext® Ultra™ DNA Library Prep Kit for Illumina® (NEB, USA) following the manufacturer's instructions and index codes were added. Then, the library was sequenced on a Illumina HiSeq 2500 platform and 300 bp paired-end reads were generated at the Novo gene. The paired-end reads were merged using FLASH (v. 1.2.71). The quality of the tags was controlled in QIIME (v. 1.7.02), meanwhile all chimeras were removed. The "Core Set" of the Greengenes database3 was used for classification, and sequences with >97% similarity were assigned to the same operational taxonomic units (OTUs).

Analysis of sequencing data Operational taxonomic unit abundance information was normalized using a standard of sequence number corresponding to the sample with the least sequences. The alpha diversity index was calculated with QIIME (v. 1.7.0). The Unifrac distance was obtained using QIIME (v. 1.7.0), and PCoA (principal coordinate analysis) was performed using R software (v. 2.15.3). The linear discriminate analysis effect size (LEfSe) was performed to determine differences in abundance; the threshold LDA score was 4.0. GraphPad Prism7 software was used to produce the graphs.

Plasma and testis metabolite measurements by LC-MS/MS

Plasma samples were collected and immediately stored at -80 °C. Before LC-MS/MS analysis, the samples were thawed on ice and processed to remove proteins. Testis samples were collected and the same amount of tissue from each mouse testis was used to isolate the metabolites using CH_3OH: H_2O (V:V) = 4:1. Then samples were analyzed by ACQUITY UPLC and AB Sciex Triple TOF 5600 (LC/MS) as reported previously [39]. Fifteen samples/group were analyzed for plasma or testis tissues.

HPLC used an ACQUITY UPLC BEH C18 column (100 mm × 2.1 mm, 1.7 μm), solvent A [aqueous solution with 0.1% (v/v) formic acid], and solvent B [acetonitrile with 0.1% (v/v) formic acid] with a gradient program:

Time	A%	B%
0	95	5
2	80	20
4	75	25
9	40	60
17	0	100
19	0	100
19.1	95	5
20.1	95	5

The flow rate was 0.4 mL/min and the injection volume was 5 μL.

The mass spectrometry program with ESI was:

Parameters	Positive ion	Positive ion
Nebulizer Gas (GS1, PSI)	40	40
Auxiliary Gas (GS2, PSI)	40	40
Curtain Gas (CUR, PSI)	35	35
Ion Source Temperature (°C)	550	550
Ion Spray Voltage (V)	5500	4500
Declustering Potential (DP,V)	100	-100
Mass Scan Range (TOF MS scan)	70–1000	70–1000
Collision Energy (TOF MS scan, eV)	10	-10
Mass Scan Range (Product Ion scan)	50–1000	50–1000
Collision Energy (Product Ion scan, eV）	30	30
Interface Heater Temperature (°C)	550	600

Progenesis QI v. 2.3 (Nonlinear Dynamics, Newcastle, UK) was used to normalize the peaks. Then the Human Metabolome Database (HMDB), Lipidmaps (v. 2.3), and METLIN software were used to qualify the data. Moreover, the data were processed with SIMCA software (v. 14.0, Umetrics, Umeå, Sweden) following pathway enrichment analysis using the KEGG database (http://www.genome.jp/KEGG/pathway.html).

Histopathological analysis

Testicular tissues were fixed in 10% neutral buffered formalin, paraffin embedded, cut into 5 μm sections and subsequently stained with hematoxylin and eosin for histopathological analysis.

Immunofluorescence staining (IHF)

The procedure for immunofluorescence staining is reported in our recent publications [58,64]. Table S8 lists the primary antibodies. Briefly, testis sections (5 μm) were prepared and subjected to antigen retrieval. Sections were then blocked with normal goat serum in PBS, followed by incubation (1:150 in PBS-1% BSA) with primary antibodies at 4 ^{o}C overnight. After a brief wash, sections were incubated with goat anti-rabbit or donkey anti-goat secondary Abs (1:100 in PBS; Beyotime Institute of Biotechnology, Shanghai, P.R. China) at RT for 30 min and then counterstained with 4',6-diamidino-2-phenylindole (DAPI). The stained sections were visualized using a Nikon Eclipse TE2000-U fluorescence microscope (Nikon, Inc., Melville, NY), and the captured fluorescence images were analyzed using MetaMorph software. Six samples/group were determined.

Western blotting

Western blotting analysis followed the procedure reported in our previous publications [58,64]. Briefly, testis tissue samples were lysed in RIPA buffer containing

a protease inhibitor cocktail from Sangong Biotech, Ltd. (Shanghai, China). Protein concentration was determined by BCA kit (Beyotime Institute of Biotechnology). Information for primary antibodies is given in Table S8. Secondary donkey anti-goat Ab (Cat no.: A0181) was purchased from Beyotime Institute of Biotechnology, and goat anti-rabbit (Cat no.: A24531) Abs were purchased from Novex® by Life Technologies (USA). Fifty micrograms of total protein per sample were loaded onto 10% SDS polyacrylamide electrophoresis gels. The gels were transferred to a polyvinylidene fluoride (PVDF) membrane at 300 mA for 2.5 h, at 4 ℃. Then, the membranes were blocked with 5% BSA for 1 h at RT, followed by three washes with 0.1% Tween-20 in TBS (TBST). The membranes were incubated with primary Abs diluted with 1:500 in TBST with 1% BSA overnight at 4 ℃. After three washes with TBST, the blots were incubated with the HRP-labelled secondary goat anti-rabbit or donkey anti-goat Abs respectively for 1 h at RT. After three washes, the blots were imaged. The experiment was performed with six individual samples/group.

Statistical analysis

Data were analyzed using SPSS statistical software (IBM Co., NY, USA) with one-way analysis of variance (ANOVA) followed by LSD multiple comparison tests. All groups were compared with each other for every parameter. The data were shown as the mean ± SEM. Statistical significance was based on $p < 0.05$.

Supplementary Materials

Figure S1. Additional data for single cell RNA seq (scRNA seq) analysis of all cells in four group samples.

Figure S2. Additional data for sub-clustering of SPGs, SPCs, and STs in single cell RNA seq (scRNA seq) analysis.

Figure S3. Heatmap of gene expression for SPG, SPC, ST, and LC/SC clusters in each group sample.

Figure S4. Enrichment analysis data for each group sampled individually.

Figure S5. GO enrichment analysis for down-regulated genes in each of the four comparisons (*ex vivo*).

Table S1. Differentially expressed genes in scRNA seq analysis for all the samples.

Table S2. Marker genes in scRNA seq analysis for all the samples.

Table S3. Motif Enrichment data for transcriptional factor analysis.

Table S4. Regulon target information in transcriptional factor analysis.

Table S5. Testis metabolites.

Table S6. Plasma metabolites.

Table S7. Correlation of testis metabolites and plasma metabolites.

Table S8. Relative amounts of microbiota in small intestine samples.

Table S9. Information for primary antibodies.

Supplementary Table 9. Primary antibody information

Gene symbol	Name	Cat. #	Predicted size	Source (Animal)	Company
DDX4 (VASA)	DEAD (Asp Glu Ala Asp) box polypeptide	ab13840	76kDa	Rabbit (polyclonal)	Abcam
DAZL	DAZ like autosomal	ab34139	33kDa	Rabbit (polyclonal)	Abcam
SCP3/SYCP3	Synaptonemal complex protein 3	NB300-232	28kDa	Rabbit (polyclonal)	Novus Biologicals
SOX9	SRY (sex-determining region Y)-box 9 protein	AB5535	65kDa	Rabbit (polyclonal)	Merck Millipore
TNP1(TR1)	Transition protein-1	ab73135		Rabbit (polyclonal)	Abcam
Jun D	Transcription factor jun-D	bs-1393R	38kDa	Rabbit (polyclonal)	Beijing Biosynthesis Biotechnology CO.
Wt1	Wilms tumor protein	bs-6983R	55kDa	Rabbit (polyclonal)	Beijing Biosynthesis Biotechnology CO.
PIWIL1	Piwi like protein 1	ab94917	99kDa	Rabbit (polyclonal)	Abcam
ZFP37	Zinc finger protein 37 homolog	bs-19125R	71kDa	Mouse(monoclonal)	Beijing Biosynthesis Biotechnology CO.
P-ERK	Phospho-Erk1 (Thr202 + Tyr204)	bs-1645R	43kDa	Rabbit (polyclonal)	Beijing Biosynthesis Biotechnology CO.
GPX1	Glutathione peroxidase 1	bs-3882R	22kDa	Rabbit (polyclonal)	Beijing Biosynthesis Biotechnology CO.
Caspase 8	Caspase 8	bs-0052R	12/55kDa	Rabbit (polyclonal)	Beijing Biosynthesis Biotechnology CO.
ACTB	Anti-ACTB rabbit polyclonal antibody	D110001-0200	42kDa	Rabbit (polyclonal)	Sangon Biotech.
actin	Actin	Ab3280	42kDa	Rabbit (polyclonal)	Abcam

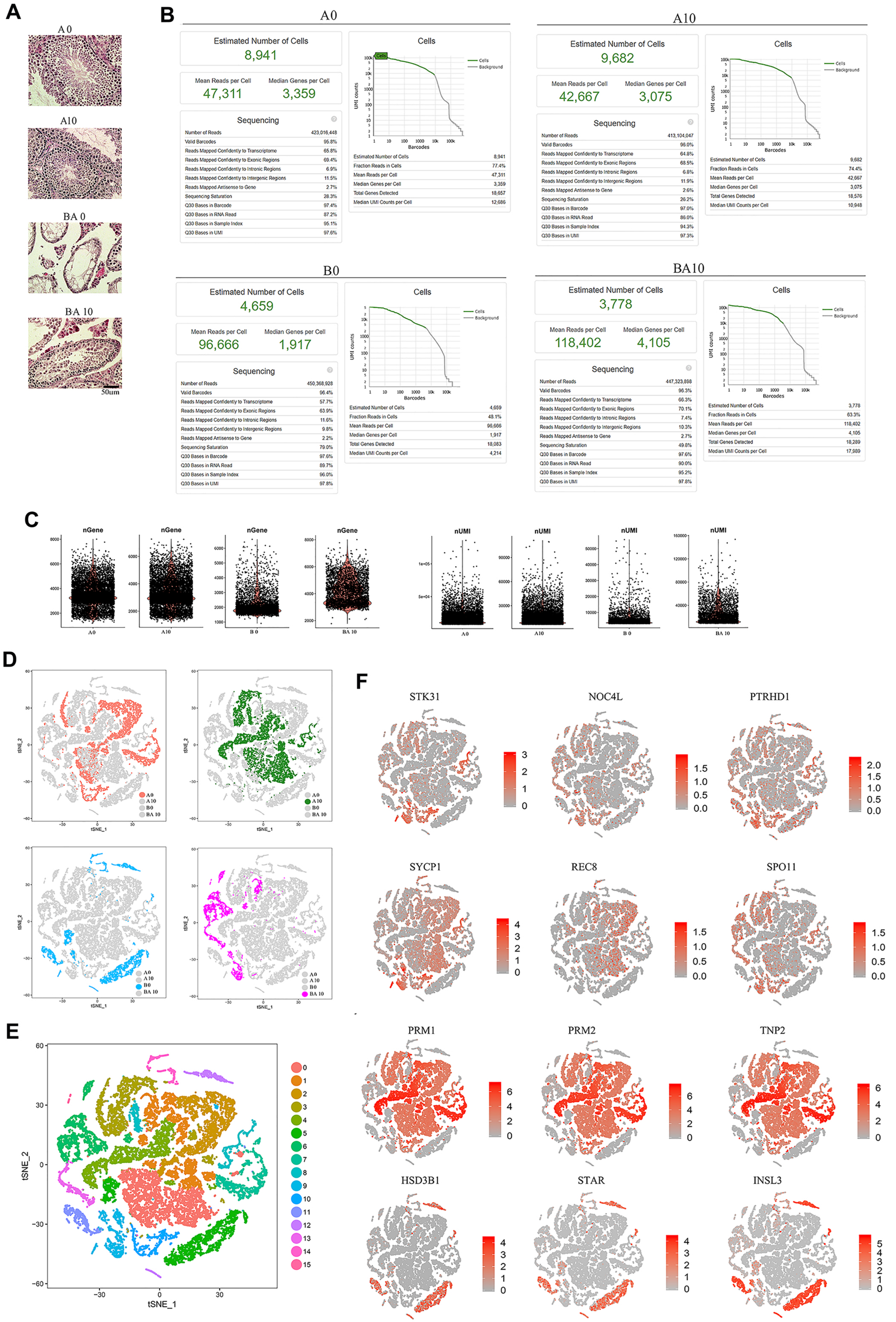

A
A 0
A10
BA 0
BA 10
50um
B
A0
Estimated Number of Cells
8,941
Mean Reads per Cell
47,311
Median Genes per Cell
3,359
Sequencing
Number of Reads 423,016,448
Valid Barcodes 95.8%
Reads Mapped Confidently to Transcriptome 65.8%
Reads Mapped Confidently to Exonic Regions 69.4%
Reads Mapped Confidently to Intronic Regions 6.9%
Reads Mapped Confidently to Intergenic Regions 11.5%
Reads Mapped Antisense to Gene 2.7%
Sequencing Saturation 28.3%
Q30 Bases in Barcode 97.4%
Q30 Bases in RNA Read 87.2%
Q30 Bases in Sample Index 95.1%
Q30 Bases in UMI 97.6%
Cells
Estimated Number of Cells 8,941
Fraction Reads in Cells 77.4%
Mean Reads per Cell 47,311
Median Genes per Cell 3,359
Total Genes Detected 18,657
Median UMI Counts per Cell 12,686
A10
Estimated Number of Cells
9,682
Mean Reads per Cell
42,667
Median Genes per Cell
3,075
Sequencing
Number of Reads 413,104,047
Valid Barcodes 96.0%
Reads Mapped Confidently to Transcriptome 64.8%
Reads Mapped Confidently to Exonic Regions 68.5%
Reads Mapped Confidently to Intronic Regions 6.8%
Reads Mapped Confidently to Intergenic Regions 11.9%
Reads Mapped Antisense to Gene 2.6%
Sequencing Saturation 26.2%
Q30 Bases in Barcode 97.0%
Q30 Bases in RNA Read 86.0%
Q30 Bases in Sample Index 94.3%
Q30 Bases in UMI 97.3%
Cells
Estimated Number of Cells 9,682
Fraction Reads in Cells 74.4%
Mean Reads per Cell 42,667
Median Genes per Cell 3,075
Total Genes Detected 18,576
Median UMI Counts per Cell 10,948
B0
Estimated Number of Cells
4,659
Mean Reads per Cell
96,666
Median Genes per Cell
1,917
Sequencing
Number of Reads 450,368,928
Valid Barcodes 96.4%
Reads Mapped Confidently to Transcriptome 57.7%
Reads Mapped Confidently to Exonic Regions 63.9%
Reads Mapped Confidently to Intronic Regions 11.6%
Reads Mapped Confidently to Intergenic Regions 9.8%
Reads Mapped Antisense to Gene 2.2%
Sequencing Saturation 79.0%
Q30 Bases in Barcode 97.6%
Q30 Bases in RNA Read 89.7%
Q30 Bases in Sample Index 96.0%
Q30 Bases in UMI 97.8%
Cells
Estimated Number of Cells 4,659
Fraction Reads in Cells 48.1%
Mean Reads per Cell 96,666
Median Genes per Cell 1,917
Total Genes Detected 18,083
Median UMI Counts per Cell 4,214
BA10
Estimated Number of Cells
3,778
Mean Reads per Cell
118,402
Median Genes per Cell
4,105
Sequencing
Number of Reads 447,323,898
Valid Barcodes 96.3%
Reads Mapped Confidently to Transcriptome 66.3%
Reads Mapped Confidently to Exonic Regions 70.1%
Reads Mapped Confidently to Intronic Regions 7.4%
Reads Mapped Confidently to Intergenic Regions 10.3%
Reads Mapped Antisense to Gene 2.7%
Sequencing Saturation 49.8%
Q30 Bases in Barcode 97.6%
Q30 Bases in RNA Read 90.0%
Q30 Bases in Sample Index 95.2%
Q30 Bases in UMI 97.8%
Cells
Estimated Number of Cells 3,778
Fraction Reads in Cells 63.3%
Mean Reads per Cell 118,402
Median Genes per Cell 4,105
Total Genes Detected 18,289
Median UMI Counts per Cell 17,989
Cells
Background
UMI counts
Barcodes
C
nGene
nUMI
A0
A10
B 0
BA 10
D
tSNE_1
tSNE_2
A0
A 10
B0
BA 10
E
tSNE_1
tSNE_2
0
1
2
3
4
5
6
7
8
9
10
11
12
13
14
15
F
STK31
NOC4L
PTRHD1
SYCP1
REC8
SPO11
PRM1
PRM2
TNP2
HSD3B1
STAR
INSL3

Figure S1. Additional data for single cell RNA seq (scRNA seq) analysis of all cells in four group samples.

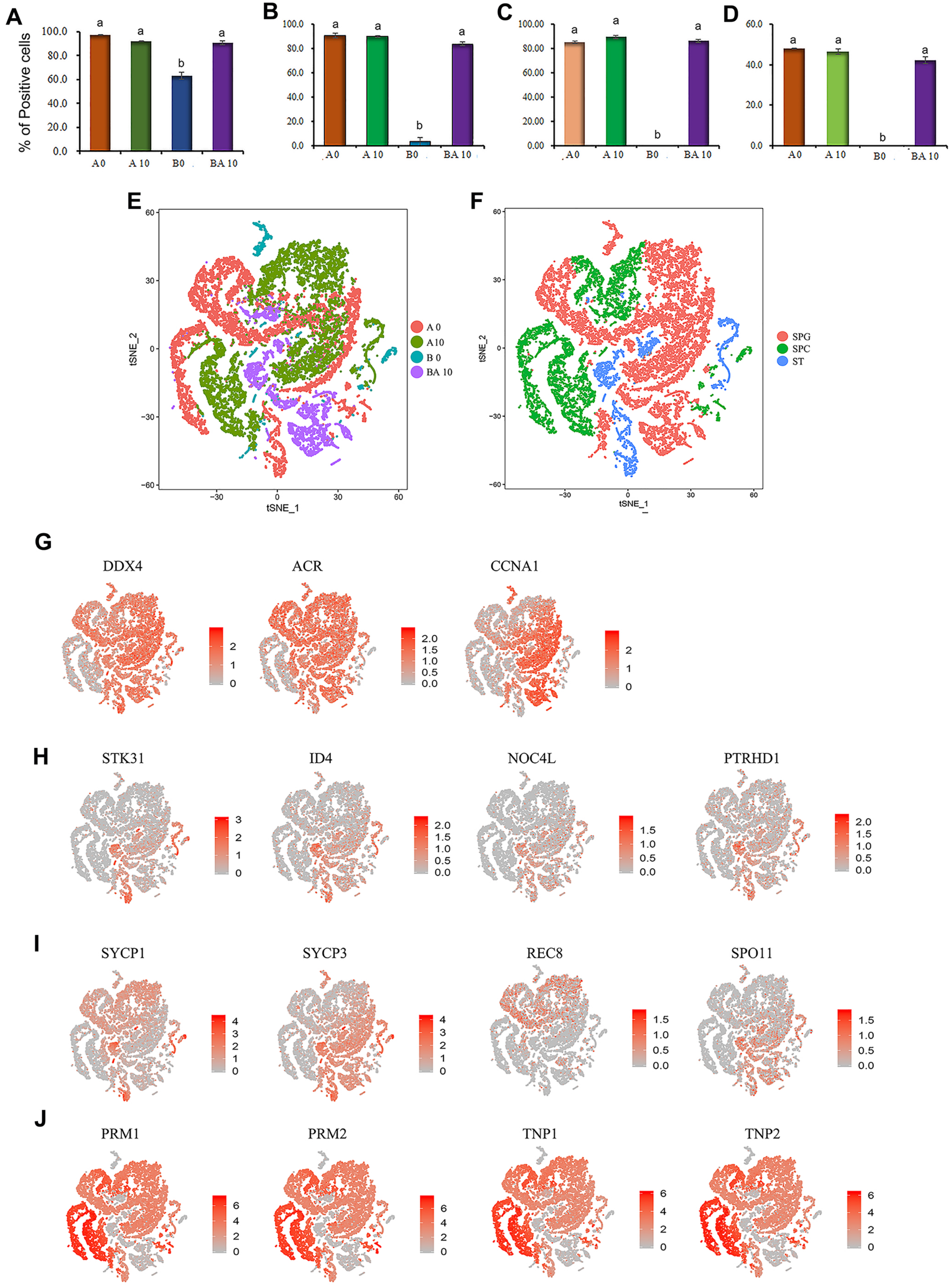

A
% of Positive cells
100.0
80.0
60.0
40.0
20.0
0.0
a
a
b
a
A 0
A 10
B0
BA 10
B
C
D
E
A 0
A10
B 0
BA 10
tSNE_1
tSNE_2
F
SPG
SPC
ST
G
DDX4
ACR
CCNA1
H
STK31
ID4
NOC4L
PTRHD1
I
SYCP1
SYCP3
REC8
SPO11
J
PRM1
PRM2
TNP1
TNP2

Figure S2. Additional data for sub-clustering of SPGs, SPCs, and STs in single cell RNA seq (scRNA seq) analysis.

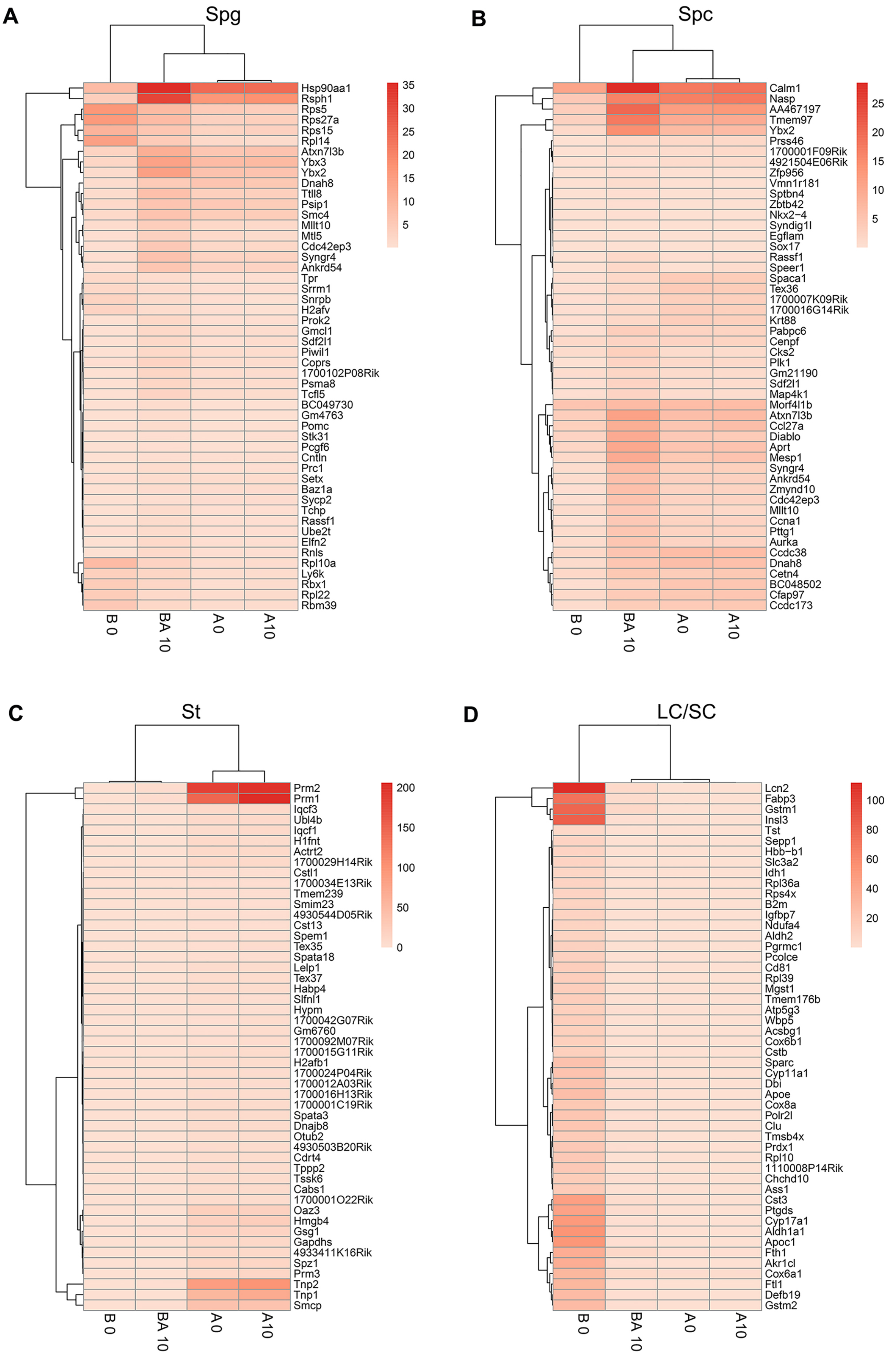

A
Spg
Hsp90aa1
Rsph1
Rps5
Rps27a
Rps15
Rpl14
Atxn7l3b
Ybx3
Ybx2
Dnah8
Ttll8
Psip1
Smc4
Mllt10
Mtl5
Cdc42ep3
Syngr4
Ankrd54
Tpr
Srrm1
Snrpb
H2afv
Prok2
Gmcl1
Sdf2l1
Piwil1
Coprs
1700102P08Rik
Psma8
Tcfl5
BC049730
Gm4763
Pomc
Stk31
Pcgf6
Cntln
Prc1
Setx
Baz1a
Sycp2
Tchp
Rassf1
Ube2t
Elfn2
Rnls
Rpl10a
Ly6k
Rbx1
Rpl22
Rbm39
B 0
BA 10
A0
A10
35
30
25
20
15
10
5
B
Spc
Calm1
Nasp
AA467197
Tmem97
Ybx2
Prss46
1700001F09Rik
4921504E06Rik
Zfp956
Vmn1r181
Sptbn4
Zbtb42
Nkx2−4
Syndig1l
Egflam
Sox17
Rassf1
Speer1
Spaca1
Tex36
1700007K09Rik
1700016G14Rik
Krt88
Pabpc6
Cenpf
Cks2
Plk1
Gm21190
Sdf2l1
Map4k1
Morf4l1b
Atxn7l3b
Ccl27a
Diablo
Aprt
Mesp1
Syngr4
Ankrd54
Zmynd10
Cdc42ep3
Mllt10
Ccna1
Pttg1
Aurka
Ccdc38
Dnah8
Cetn4
BC048502
Cfap97
Ccdc173
B 0
BA 10
A0
A10
25
20
15
10
5
C
St
Prm2
Prm1
Iqcf3
Ubl4b
Iqcf1
H1fnt
Actrt2
1700029H14Rik
Cstl1
1700034E13Rik
Tmem239
Smim23
4930544D05Rik
Cst13
Spem1
Tex35
Spata18
Lelp1
Tex37
Habp4
Slfnl1
Hypm
1700042G07Rik
Gm6760
1700092M07Rik
1700015G11Rik
H2afb1
1700024P04Rik
1700012A03Rik
1700016H13Rik
1700001C19Rik
Spata3
Dnajb8
Otub2
4930503B20Rik
Cdrt4
Tppp2
Tssk6
Cabs1
1700001O22Rik
Oaz3
Hmgb4
Gsg1
Gapdhs
4933411K16Rik
Spz1
Prm3
Tnp2
Tnp1
Smcp
B 0
BA 10
A0
A10
200
150
100
50
0
D
LC/SC
Lcn2
Fabp3
Gstm1
Insl3
Tst
Sepp1
Hbb−b1
Slc3a2
Idh1
Rpl36a
Rps4x
B2m
Igfbp7
Ndufa4
Aldh2
Pgrmc1
Pcolce
Cd81
Rpl39
Mgst1
Tmem176b
Atp5g3
Wbp5
Acsbg1
Cox6b1
Cstb
Sparc
Cyp11a1
Dbi
Apoe
Cox8a
Polr2l
Clu
Tmsb4x
Prdx1
Rpl10
1110008P14Rik
Chchd10
Ass1
Cst3
Ptgds
Cyp17a1
Aldh1a1
Apoc1
Fth1
Akr1cl
Cox6a1
Ftl1
Defb19
Gstm2
B 0
BA 10
A0
A10
100
80
60
40
20

Figure S3. Heatmap of gene expression for SPG, SPC, ST, and LC/SC clusters in each group sample.

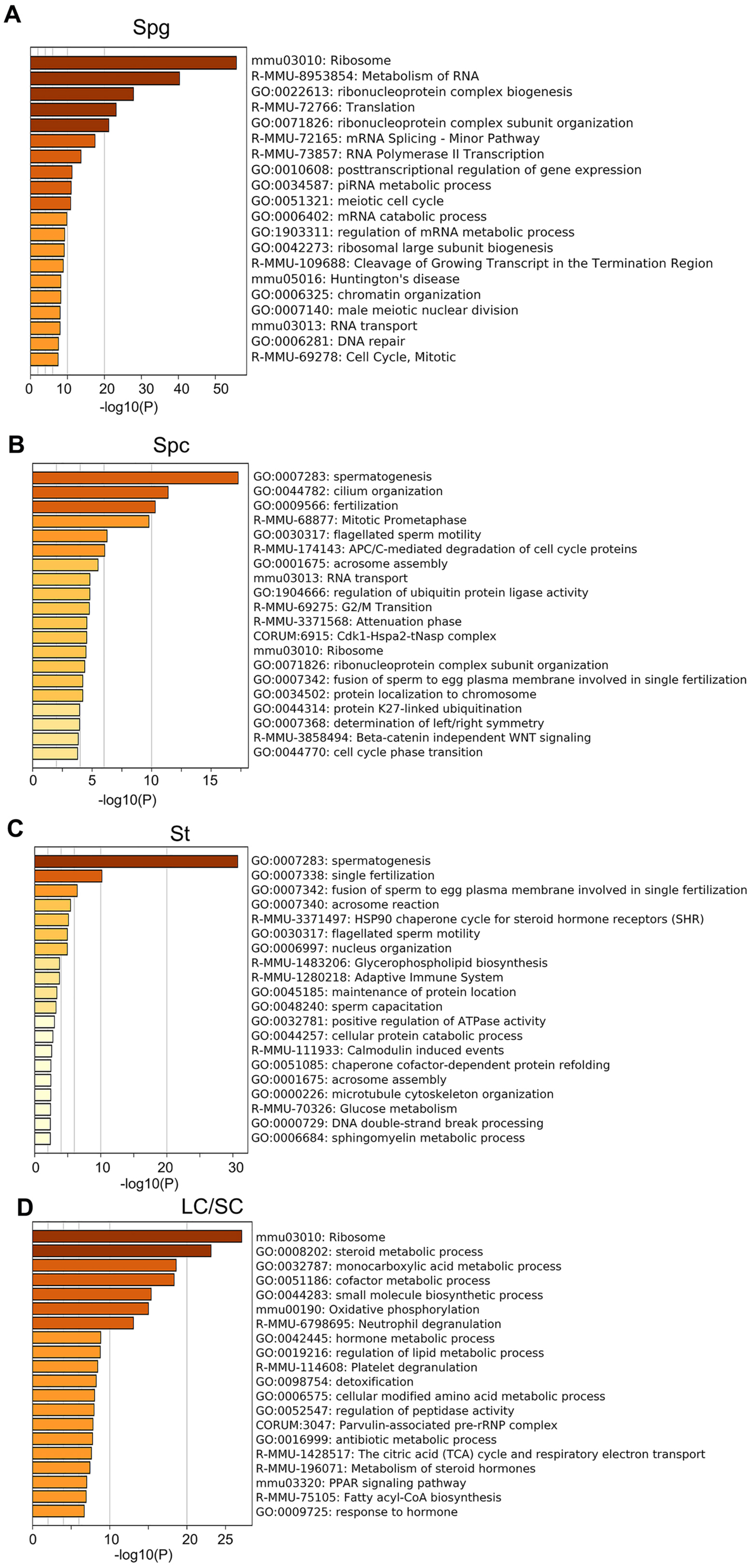
A
Spg
mmu03010: Ribosome
R-MMU-8953854: Metabolism of RNA
GO:0022613: ribonucleoprotein complex biogenesis
R-MMU-72766: Translation
GO:0071826: ribonucleoprotein complex subunit organization
R-MMU-72165: mRNA Splicing - Minor Pathway
R-MMU-73857: RNA Polymerase II Transcription
GO:0010608: posttranscriptional regulation of gene expression
GO:0034587: piRNA metabolic process
GO:0051321: meiotic cell cycle
GO:0006402: mRNA catabolic process
GO:1903311: regulation of mRNA metabolic process
GO:0042273: ribosomal large subunit biogenesis
R-MMU-109688: Cleavage of Growing Transcript in the Termination Region
mmu05016: Huntington's disease
GO:0006325: chromatin organization
GO:0007140: male meiotic nuclear division
mmu03013: RNA transport
GO:0006281: DNA repair
R-MMU-69278: Cell Cycle, Mitotic
0
10
20
30
40
50
-log10(P)
B
Spc
GO:0007283: spermatogenesis
GO:0044782: cilium organization
GO:0009566: fertilization
R-MMU-68877: Mitotic Prometaphase
GO:0030317: flagellated sperm motility
R-MMU-174143: APC/C-mediated degradation of cell cycle proteins
GO:0001675: acrosome assembly
mmu03013: RNA transport
GO:1904666: regulation of ubiquitin protein ligase activity
R-MMU-69275: G2/M Transition
R-MMU-3371568: Attenuation phase
CORUM:6915: Cdk1-Hspa2-tNasp complex
mmu03010: Ribosome
GO:0071826: ribonucleoprotein complex subunit organization
GO:0007342: fusion of sperm to egg plasma membrane involved in single fertilization
GO:0034502: protein localization to chromosome
GO:0044314: protein K27-linked ubiquitination
GO:0007368: determination of left/right symmetry
R-MMU-3858494: Beta-catenin independent WNT signaling
GO:0044770: cell cycle phase transition
0
5
10
15
-log10(P)
C
St
GO:0007283: spermatogenesis
GO:0007338: single fertilization
GO:0007342: fusion of sperm to egg plasma membrane involved in single fertilization
GO:0007340: acrosome reaction
R-MMU-3371497: HSP90 chaperone cycle for steroid hormone receptors (SHR)
GO:0030317: flagellated sperm motility
GO:0006997: nucleus organization
R-MMU-1483206: Glycerophospholipid biosynthesis
R-MMU-1280218: Adaptive Immune System
GO:0045185: maintenance of protein location
GO:0048240: sperm capacitation
GO:0032781: positive regulation of ATPase activity
GO:0044257: cellular protein catabolic process
R-MMU-111933: Calmodulin induced events
GO:0051085: chaperone cofactor-dependent protein refolding
GO:0001675: acrosome assembly
GO:0000226: microtubule cytoskeleton organization
R-MMU-70326: Glucose metabolism
GO:0000729: DNA double-strand break processing
GO:0006684: sphingomyelin metabolic process
0
10
20
30
-log10(P)
D
LC/SC
mmu03010: Ribosome
GO:0008202: steroid metabolic process
GO:0032787: monocarboxylic acid metabolic process
GO:0051186: cofactor metabolic process
GO:0044283: small molecule biosynthetic process
mmu00190: Oxidative phosphorylation
R-MMU-6798695: Neutrophil degranulation
GO:0042445: hormone metabolic process
GO:0019216: regulation of lipid metabolic process
R-MMU-114608: Platelet degranulation
GO:0098754: detoxification
GO:0006575: cellular modified amino acid metabolic process
GO:0052547: regulation of peptidase activity
CORUM:3047: Parvulin-associated pre-rRNP complex
GO:0016999: antibiotic metabolic process
R-MMU-1428517: The citric acid (TCA) cycle and respiratory electron transport
R-MMU-196071: Metabolism of steroid hormones
mmu03320: PPAR signaling pathway
R-MMU-75105: Fatty acyl-CoA biosynthesis
GO:0009725: response to hormone
0
10
20
25
-log10(P)

Figure S4. Enrichment analysis data for each group sampled individually.

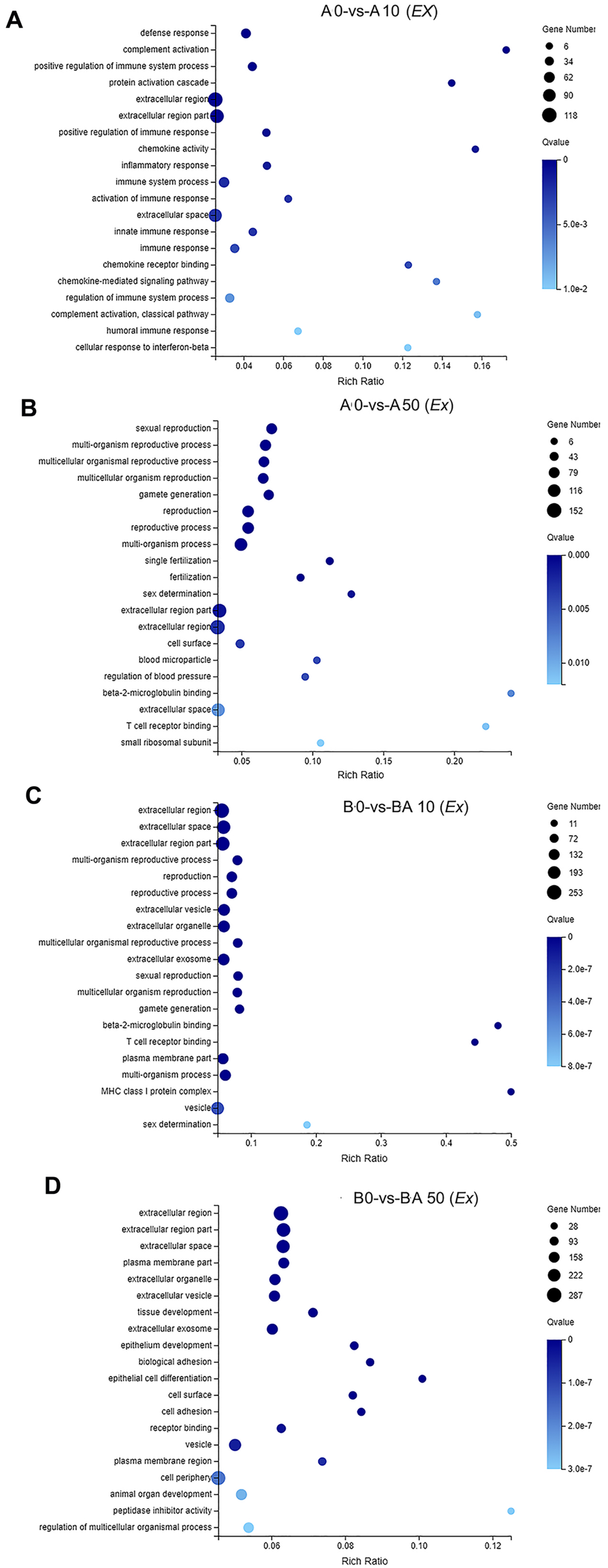
A
A0-vs-A10 (EX)
defense response
complement activation
positive regulation of immune system process
protein activation cascade
extracellular region
extracellular region part
positive regulation of immune response
chemokine activity
inflammatory response
immune system process
activation of immune response
extracellular space
innate immune response
immune response
chemokine receptor binding
chemokine-mediated signaling pathway
regulation of immune system process
complement activation, classical pathway
humoral immune response
cellular response to interferon-beta
Rich Ratio
Gene Number
Qvalue
B
A0-vs-A50 (Ex)
sexual reproduction
multi-organism reproductive process
multicellular organismal reproductive process
multicellular organism reproduction
gamete generation
reproduction
reproductive process
multi-organism process
single fertilization
fertilization
sex determination
extracellular region part
extracellular region
cell surface
blood microparticle
regulation of blood pressure
beta-2-microglobulin binding
extracellular space
T cell receptor binding
small ribosomal subunit
Rich Ratio
C
B0-vs-BA 10 (Ex)
extracellular region
extracellular space
extracellular region part
multi-organism reproductive process
reproduction
reproductive process
extracellular vesicle
extracellular organelle
multicellular organismal reproductive process
extracellular exosome
sexual reproduction
multicellular organism reproduction
gamete generation
beta-2-microglobulin binding
T cell receptor binding
plasma membrane part
multi-organism process
MHC class I protein complex
vesicle
sex determination
Rich Ratio
D
B0-vs-BA 50 (Ex)
extracellular region
extracellular region part
extracellular space
plasma membrane part
extracellular organelle
extracellular vesicle
tissue development
extracellular exosome
epithelium development
biological adhesion
epithelial cell differentiation
cell surface
cell adhesion
receptor binding
vesicle
plasma membrane region
cell periphery
animal organ development
peptidase inhibitor activity
regulation of multicellular organismal process
Rich Ratio

Figure S5. GO enrichment analysis for down-regulated genes in each of the four comparisons (*ex vivo*).
